# A Humanized Mouse Model of HPV-Associated Pathology Driven by E7 Expression

**DOI:** 10.1371/journal.pone.0041743

**Published:** 2012-07-23

**Authors:** Águeda Buitrago-Pérez, Mariam Hachimi, Marta Dueñas, Belén Lloveras, Almudena Santos, Almudena Holguín, Blanca Duarte, Juan Luis Santiago, Baki Akgül, José L. Rodríguez-Peralto, Alan Storey, Catalina Ribas, Fernando Larcher, Marcela del Rio, Jesús M. Paramio, Ramón García-Escudero

**Affiliations:** 1 Molecular Oncology Unit, CIEMAT, Madrid, Spain; 2 Hospital del Mar, Barcelona, Spain; 3 Departamento de Biología Molecular and Centro de Biología Molecular Severo Ochoa, CSIC-UAM, Universidad Autónoma de Madrid, Madrid, Spain; 4 Instituto de Investigación Sanitaria La Princesa, Madrid, Spain; 5 Regenerative Medicine Unit, Division of Biomedicine, CIEMAT, Madrid, Spain; 6 Cutaneous Diseases Modeling Unit, Division of Biomedicine, CIEMAT, Madrid, Spain; 7 Departamento de Dermatología, Hospital General de Ciudad Real, Universidad de Castilla - La Mancha, Ciudad Real, Spain; 8 Institute of Virology, University of Cologne, Cologne, Germany; 9 Pathology Department, Hospital Universitario 12 de Octubre, and Instituto de Investigación Hospital 12 de Octubre i+12, Universidad Complutense, Madrid, Spain; 10 Department of Oncology, University of Oxford, John Radcliffe Hospital, Headington, Oxford, United Kingdom; IPO, Inst Port Oncology, Portugal

## Abstract

Human papillomavirus (HPV) is the causative agent of human cervical cancer and has been associated with oropharyngeal squamous cell carcinoma development. Although prophylactic vaccines have been developed, there is a need to develop new targeted therapies for individuals affected with malignant infected lesions in these locations, which must be tested in appropriate models. Cutaneous beta HPV types appear to be involved in skin carcinogenesis. Virus oncogenicity is partly achieved by inactivation of retinoblastoma protein family members by the viral E7 gene. Here we show that the E7 protein of cutaneous beta HPV5 binds pRb and promotes its degradation. In addition, we described an *in vivo* model of HPV-associated disease in which artificial human skin prepared using primary keratinocytes engineered to express the E7 protein is engrafted onto nude mice. Expression of E7 in the transplants was stably maintained for up to 6 months, inducing the appearance of lesions that, in the case of HPV16 E7, histologically resembled human anogenital lesions caused by oncogenic HPVs. Moreover, it was confirmed through biomarker expression analysis via immunodetection and/or quantitative PCR from mRNA and miRNA that the 16E7-modified engrafted skin shares molecular features with human HPV-associated pretumoral and tumoral lesions. Finally, our findings indicate a decrease of the *in vitro* capacity of HPV5 E7 to reduce pRb levels *in vivo*, possibly explaining the phenotypical differences when compared with 16E7-grafts. Our model seems to be a valuable platform for basic research into HPV oncogenesis and preclinical testing of HPV-associated antitumor therapies.

## Introduction

Human papillomaviruses (HPVs) are small DNA viruses that show strict tropism for squamous epithelium, such as cutaneous epidermis or genital and oral mucosas. Certain HPV types are related with human cancer of the skin, anogenital region, and oropharynx [1]–[4]. The link between cervical cancer (CC), the second most frequent cancer in women worldwide, and HPVs was first described in 1983 by Harald zur Hausen [5]. Today there is overwhelming evidence that certain high-risk (HR) types of mucosal HPVs cause CC. Indeed, HPV types 16 and 18 are responsible for around 70% of CC worldwide [6]. In recent decades there has been a notable increase in the number of cases detected of HPV-positive cancers of the tonsils and base of the tongue and of oropharyngeal squamous cell carcinoma (OSCC). Importantly, HPV-positive OSCC shows a significantly better response to therapy than HPV-negative OSCC [7]–[12]. The association between cutaneous HPV and skin cancer was first suspected in patients with the rare inherited disease epidermodysplasia verruciformis (EV). These patients are highly susceptible to infection with betapapillomavirus (betaPV) types (such as HPV5 and 8) and frequently develop keratotic skin lesions and squamous cell carcinomas (SCC) of the skin [13], [14]. In addition, the presence of betaPV DNA in a high proportion of skin SCCs and actinic keratoses (AK) has been observed in organ transplant recipients (OTRs) [15] and immunocompetent individuals [16]. Moreover, the prevalence of betaPV infection in patients with SCC exceeds 80% [17]. However, it should be mentioned that betaPV types show a similar prevalence in normal skin and a very low viral load in AK and SCC. Although some reports suggest that betaPV may play a role in the development of cutaneous SCC [18], epidemiological studies have not clearly established a direct link between HPV infection and non-melanoma skin cancer.

HPV infects the basal layer of the squamous epithelium, where the viral DNA persists for many years in an episomal form [19]. The virus life cycle depends on the differentiation status of the host cell, as mature particles are released upon epidermal desquamation [4], [20]. As virus replication requires the host cell’s machinery, HPV induces the expression of host DNA replication proteins in terminally differentiated post-mitotic cells by disrupting the strict coupling between keratinocyte cell cycle arrest and differentiation [21]. Cellular regulators of these processes are the retinoblastoma protein (pRb) and the p53 tumor suppressors, which are inhibited by the HR-HPV E7 and E6 viral oncogenes, respectively [22]–[24]. HPV16-derived E7 protein binds to the 3 members of the retinoblastoma protein family: pRb, p107 and p130. Upon interaction, these proteins are targeted for degradation [25], [26], allowing the expression of genes involved in G1-S transition, thereby cell proliferation. Mouse models have demonstrated that E7 is the most important HPV16 oncogene for oral and cervical carcinogenesis, and this property is partially mediated by the inactivation of pRb [27], [28].

The molecular transforming activities of betaPVs in skin cancer have not yet been fully characterized. Some betaPVs are thought to act by potentiating the harmful effects of UV radiation, for example, by impairing DNA repair and apoptosis following UV-induced damage through E6 molecular activities [29]–[34]. Although the interaction between E7 proteins from betaPVs with pRb has already been described [35], [36], inhibition of the pocket proteins has not been fully addressed for viruses such as HPV5 or 8. In addition, beta HPV38 E6 and E7 proteins are able to alter the functions of p53 and pRb by promoting p53 phosphorylation and stabilization, leading to the accumulation of ΔNp73, a potent inhibitor of p53 transcriptional functions [36], [37]. Finally, the involvement of betaPV in skin cancer development and the contribution of UV radiation has been confirmed in transgenic mice such as those expressing HPV8 or HPV38 genes [8], [38].

Although prophylactic vaccines developed against HR mucosal HPVs may protect from CC development, they are not effective in subjects who have been already infected [39]. Thus, new therapies are needed for this population at risk of carcinoma development. To pursue this goal, appropriate *in vivo* model systems are essential to examine HPV oncogenesis, to improve existing knowledge of cell targets and biomarkers of HPV-infected tumors, and to allow preclinical testing of such therapies. Previously we described a humanized animal model system based on the grafting of a human skin equivalent in *nu/nu* immunodeficient mice that has been used in clinics or for permanent skin regeneration in burn patients [40], [41]. Although the mice lack a proper immune system, the model is able to simulate physiological processes such as wound healing in a human context [42], [43]. Our system has also proved efficient for modeling inherited skin diseases including different forms of epidermolysis bullosa and testing gene therapy approaches for these diseases [44], [45].

The present study was designed to examine the molecular activities of cutaneous beta HPV5 E7 protein in relation to the retinoblastoma protein. Using the mouse human skin graft model, we characterized the long-term molecular and phenotypic consequences of E7 expression of HPV5 and HPV16. Our findings validate the use of this model for investigating HPV-associated diseases.

## Materials and Methods

### Ethics Statement

Human foreskin samples from Caucasian children donors undergoing circumcision surgery were obtained at the blood and tissue bank *Centro Comunitario de Sangre y Tejidos de Asturias*. Written informed consent was provided by next of kin, carers or guardians on the behalf of the donors. The use of this tissue was approved by the ethics committee of the tissue bank. The study protocol including the use of formalin fixed, paraffin-embedded (FFPE) blocks of human clinical samples (cervical intraepithelial neoplasia and bowenoid papulosis) was approved by the Review Boards of the Hospital del Mar in Barcelona (Spain) and Hospital Universitario 12 de Octubre in Madrid (Spain). All work was conducted according to the principles of the Declaration of Helsinki. Commercially available tissue microarrays (Cybrdi Inc., Rockville, MD, USA) were used to analyze human CC samples. Mice were kept for the duration of the experiment in pathogen-free conditions in individually ventilated type II cages (25 air changes per hour) with 10 KGy-irradiated soft wood pellets as bedding at the animal house of the CIEMAT. This institution approved all animal experimental procedures (Approval ID# 28079-21A of the Ministerio de Medio Ambiente, Medio Rural y Marino) according to Spanish and European directives.

### E7 Plasmid Vectors and Construction of Mutants

E7 genes were cloned and fused to sequences encoding Flag epitopes. Two complementary oligonucleotides (Supplementary Table S1) were hybridized and cloned into the EcoRI site of pcDNA3.1(−) (Invitrogen) containing the Flag sequence that ended with a stop codon to obtain pcDNA3.1(−)-FLAG. E7 genes from HPV10, HPV5 and HPV16 were obtained by PCR using specific primers (Table S1) from plasmids pcDNA3-10E7, pBP-5E7 and pBP-16E7, respectively. The primers allowed the cloning into pcDNA3.1(−)-FLAG upon digestion with NotI/EcoRI (10E7) or EcoRI (5E7 and 16E7) enzymes. Reverse primers lack the stop codon, enabling the epitope to be inserted in-frame at the C-terminal end of E7. Resultant E7-Flag fusions were verified by DNA sequencing. E7 mutants lacking the LxCxE motif (ΔDLYC_16E7 and ΔDLFC_5E7) were obtained using Quick Change Site-Directed Mutagenesis (Stratagene). To purify the E7 proteins, we cloned E7-flagged genes into pGEX-2T (GE Healthcare) from the corresponding pcDNA3.1(−)-E7FLAG plasmids to obtain glutathione-S-transferase (GST)-E7-Flag proteins. Thus, 10E7-Flag, 5E7-Flag and 16E7-Flag fragments were extracted after digestion with EcoRV/PmeI and cloned into pGEX-2T linearized with SmaI. The GST-E7-Flag genes were sequenced to check that the fusions were properly inserted. Retroviral transductions of 5E7-Flag and 16E7-Flag genes were performed with the 5E7-Flag and 16E7-Flag fragments extracted from the corresponding pcDNA3.1(−)-E7FLAG plasmids upon digestion with PmeI, and cloned into pLZR-IRES-GFP [46] linearized with XhoI/PmeI. Ligation clones were sequenced for adequate insertion of E7-flagged genes in the retroviral vector. pLZRS-E7-IRES-eGFP plasmids drive the expression of transgenes from the Moloney murine leukemia virus LTR sequence. The IRES sequence located between E7 and eGFP allows translation of both proteins from a common transcript, whereby E7 is produced from a 5′-capped mRNA and eGFP from an IRES-dependent translation mechanism.

### GST-E7 Fusion Protein Purification and Protein Interaction Assays

BL21 (DE3) pLysS (Promega) cells were transformed with the pGEX-2T-derived plasmids described above. Bacterial cultures were grown per each fusion protein. GST proteins were present in the supernatant of cell lysates. Purification was conducted with glutathione sepharose 4B (GE Healthcare) using the automated system Profinia™ (Bio-Rad).

Protein interaction experiments were performed using cell lysates or purified recombinant proteins. Lysates of HaCaT cells were obtained in an extraction buffer (20 mM Tris HCl pH = 7.5, 0.6 mM EDTA, 70 mM NaCl, 0.1% NP-40 and protease inhibitors). Purified His-pRb protein was purchased from Ozyme (France). Briefly, GST proteins (20 µg) were incubated with 400 µg of HaCaT lysates or 0.5 µg of recombinant His-pRb in binding buffer (20 mM Tris HCl pH = 7.5, 0.6 mM EDTA, 70 mM NaCl, 0.01% NP-40 and protease inhibitors) overnight at 4°C. Subsequently, glutathione sepharose 4B resin was added for 2 h at 4°C, after which the affinity matrix was pelleted and washed five times with 500 µl of ice-cold binding buffer. Proteins retained on the matrix were resolved by SDS-PAGE and transferred onto nitrocellulose membranes for immunoblotting.

### Cell Transfections, Retroviral Transductions and Artificial Skin Preparation

The pRb-deficient human osteosarcoma Saos2 cell line was obtained from the ATCC, and the HaCaT human skin keratinocyte cell line was a generous gift from Dr. P Boukamp (DKFZ Heidelberg, Germany). Cells were maintained in Dulbecco’s modified Eagle’s medium (DMEM) containing 10% fetal bovine serum (FBS), 1% antibiotic-antimycotic mix (Gibco), and incubated at 37°C in a humid atmosphere containing 5% CO_2_. Cotransfections to analyze pRb protein levels were performed in Saos2 cells using Fugene HD (Roche) in 10-cm dishes. Protein levels were analyzed by immunoblotting 48 hours post-transfection. Three plasmids were used: i) 7.8 µg of either pcDNA3.1(−)-5E7FLAG, or pcDNA3.1(−)-16E7FLAG or pcDNA3.1(−)-FLAG (the latter, for non-E7 control “vector” samples); ii) 2.6 µg of pcDNA-mycRb; and iii) 0.65 µg of pcDNA-eGFP. The ratio Fugene HD (µl):transfected DNA (µg) that we have used is 4∶1. In experiments where increasing amounts of E7-plasmids were compared, total cotransfected DNA was maintained constant by adding pcDNA3.1(−)-FLAG. MG132 experiments were performed by adding the inhibitor 4 hours before harvesting the Saos2 cells. Foreskin primary human keratinocytes (PHKs) and fibroblasts were obtained according to previously described methods [47]. PHKs and fibroblasts were cultured as described [47], [48]. Amphotropic retroviruses were generated by transient transfection in 293T cells as described elsewhere [46], [49]. Foreskin PHKs, seeded on lethally irradiated 3T3-fibroblast feeders, were subjected to two rounds of infection with the retroviruses. Proportions of eGFP-positive cells were determined by flow cytometry (Becton Dickinson). Aliquots of the infected cells were harvested for protein or RNA expression, or used to perform skin grafts.

A fibrin matrix populated with fibroblasts was used as the dermal component of the artificial skin. The fibrin matrix was prepared according to a procedure previously described [40], [48], [50]. Organotypic cultures were grown submerged until reaching keratinocyte confluence. At this point cultures were manually detached from the plate and placed orthotopically on the backs of immunodeficient *nu/nu* mice. The grafts were about 10×10 cm. In the intact xenograft, green fluorescence was readily visualized *in vivo* using a fluorescence stereomicroscope under blue light (Olympus America, Melville, NY). Successful engraftment mice were injected intraperitoneally with 100 µg of BrdU 1 hour before sacrifice by CO_2_ inhalation. The regenerated human skin grafts were excised along with approximately 2 mm of surrounding mouse skin. Part of the graft was immediately snap frozen in liquid nitrogen, another part was submerged in RNAlater for genetic analysis, and the remainder was placed in 4% buffered formalin or 4% paraformaldehyde, and embedded in paraffin for hematoxylin–eosin (H&E) staining or immunostaining with specific antibodies.

To generate bioengineered skin and graft it onto the backs of *nude* mice, we performed 3 sets of retroviral infections. The overall proportion of infected cells was 46% ±10% as determined by flow cytometry of eGFP positive cells (Fig. S1). Grafts were maintained for 3 to 6 months to analyze the long-term phenotypic effects and stability of viral oncogene expression. In total, four different grafts per retroviral construct (empty vector control, HPV5 E7 and HPV16 E7 recombinants) were performed per experiment set, and the experiments were repeated three times thus yielding 3 sets of 12 transplants (n = 36). Two sets were maintained for about 3 months and one set for 6 months.

### Gene Expression Analysis

For quantitative real-time PCR (qRT-PCR), total RNA including miRNA was purified using the miRNAeasy Mini Kit (Qiagen). Skin transplants were disrupted and homogenized using MixerMill 301 (Retsch). RNA integrity was tested using Bioanalyzer (Agilent). For gene expression analysis, reverse transcription was conducted with the Omniscript® Reverse Transcription kit (Qiagen) using oligo-dT primers. Real-time PCR was performed using gene specific primers (Table S1) and the SYBR Green system (Applied Biosystems). The housekeeping gene *ß-glucuronidase* (GUSB) was used for normalization. TaqMan® MicroRNA Assays (Applied Biosystems) with the TaqMan® Universal PCR Master Mix reagent kit (Applied Biosystems) were used to quantify miRNAs following the manufacturer instructions. miRNA levels were normalized using U6B as a control small RNA. qRT-PCR for mRNA and miRNA was performed in an ABI 7500fast Real-Time PCR System (Applied Biosystems). Relative expression levels were obtained by calculating E^Ct^ values (E = efficiency of PCR amplification, Ct = cycle number at the threshold level of log-based fluorescence) values, and normalized to the housekeeping RNA. Normalized expression values were log_2_ transformed, and z-scores were calculated (mean = 0; stdv = 1) per gene or miRNA.

Gene expression patterns of HPV-infected human samples of cervical cancer or vulvar intraepithelial tissues were downloaded from the Oncomine Gene Expression Signatures database [51], and previously reported using transcriptome microarray analyses [52], [53]. As for qRT-PCR, normalized microarray expression values were log_2_ transformed, and z-scores were calculated (mean = 0; stdv = 1) for E2F1, CENPF, MELK and RFC4 genes.

### Immunostaining of Paraffin Embedded Grafted Tissue

Immunohistochemistry or immunofluorescence analyses were performed as previously reported in 5 µm thick sections after fixation [54], [55]. For immunohistochemistry, the signal was amplified using avidin-peroxidase (ABC elite kit, Vector) and peroxidase was visualized using diaminobenzidine as a substrate (DAB kit, Vector). Controls were routinely performed replacing primary antibodies with PBS (data not shown). For immunofluorescence, species-specific secondary antibodies coupled to convenient fluorochromes (Jackson Immunoresearch) were used as described [56]. When necessary, DAPI (Roche) was added to identify the nuclei. BrdU detection was done with an anti-BrdU specific antibody. Immunofluorescence was observed using a microscope (Zeiss Axioplan2 imaging) equipped with an epifluorescence source and adequate filters. Images were captured with a digital camera (AxioCam MRm) and visualized using AxioVision Rel.4.6 software. The CINtec Histology Kit (mtm Laboratories, Heidelberg, Germany) was used for p16 immunohistochemistry. The primary antibodies used are listed in Table S2.

### Protein Immunoblots

Proteins were extracted from cultured cells or skin grafts after lysis using using RIPA buffer (Trizma-HCl 50 mM, NaCl 150 mM, 0.1% SDS, 1% Triton X-100 and 0.5% deoxycholate) supplemented with protease inhibitors. Protein concentration were determined by the Bradford method (BioRad). Protein separation was done using the precasted 4–12% gradient SDS-PAGE system (Invitrogen). Proteins were transferred to nitrocellulose membranes and incubated with primary antibodies. Peroxidase-coupled secondary antibodies were detected using the Super Signal West Pico Chemiluminescent Substrate (Pierce) following the manufacturer recommendations. The primary antibodies used are listed in Table S2. Protein bands were quantified using Quantity One software (Bio-Rad).

## Results

### Cutaneous HPV5 E7 Protein Reduces pRb Protein Levels

**Figure 1:**
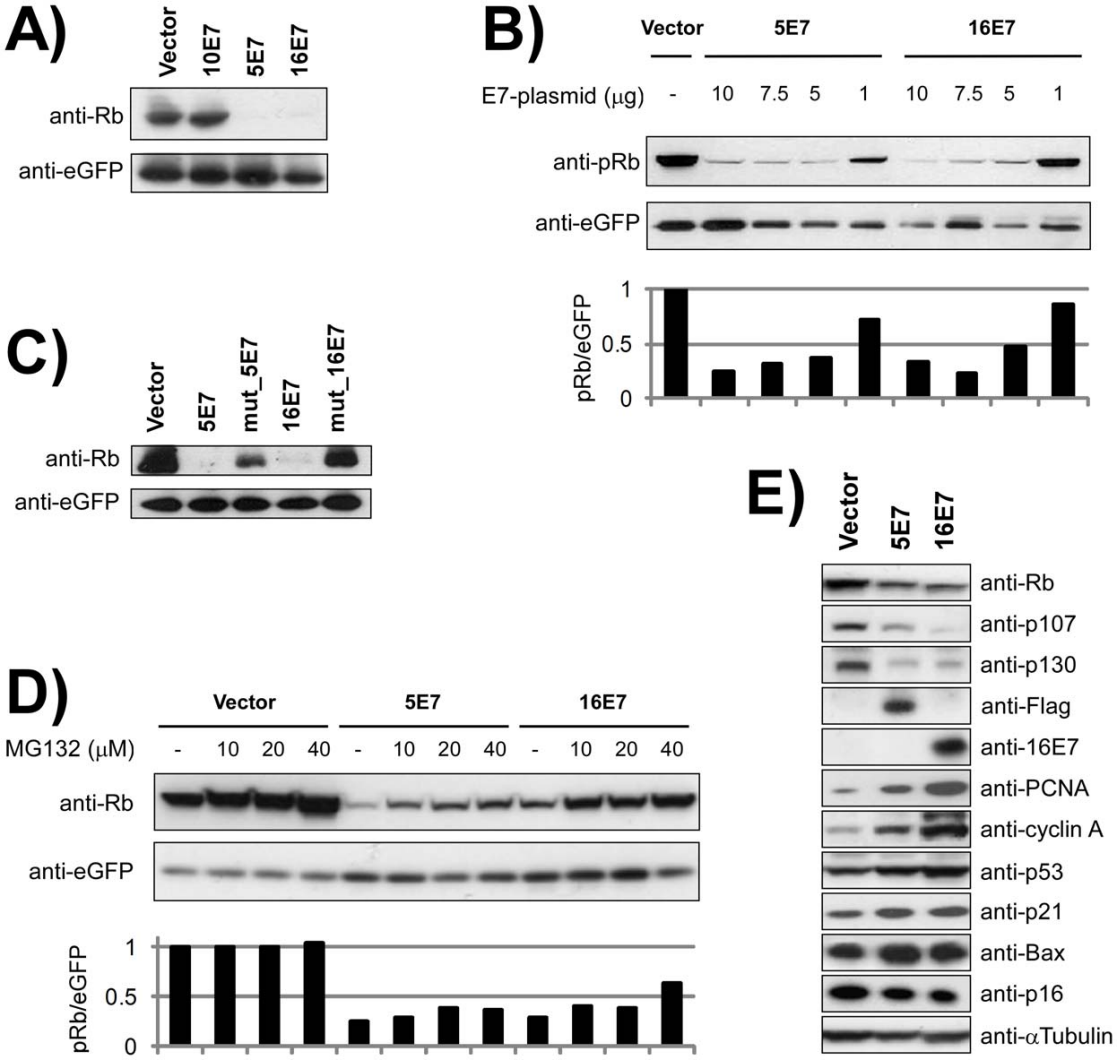
Cutaneous HPV5 E7 protein reduces pocket proteins levels. (**A**) Immunoblots of Saos2 lysates cotransfected with either pcDNA3.1(−)-FLAG (**Vector**), pcDNA3.1(−)-10E7FLAG (**10E7**), pcDNA3.1(−)-5E7FLAG (**5E7**), or pcDNA3.1(−)-16E7FLAG (**16E7**) plasmids. The effect on pRb expression levels was tested upon cotransfection with the pcDNA-mycRb vector in all cases. Transfection efficiencies were assesed using pcDNA-eGFP. Note that both 5E7 and 16E7, but not 10E7, induced a significant decrease in transiently expressed pRb protein. (**B**) Immunoblots of Saos2 lysates cotransfected with pcDNA3.1(−)-FLAG (**Vector**) or with increasing amounts of either pcDNA3.1(−)-5E7FLAG (**5E7**) or pcDNA3.1(−)-16E7FLAG (**16E7**) plasmids in the presence of similar amounts of pcDNA-mycRb. Lower panel shows the relative quantification of pRb with respect to eGFP. Note that both 5E7 and 16E7 reduced pRb levels in a dose-dependent manner. (**C**) Immunoblots of Saos2 lysates cotransfected with either pcDNA3.1(−)-FLAG (**Vector**), pcDNA3.1(−)-5E7FLAG (**5E7**), pcDNA3.1(−)-ΔDLFC_5E7FLAG (**mut_5E7**) pcDNA3.1(−)-ΔDLYC_16E7FLAG (**mut_16E7**) or pcDNA3.1(−)-16E7FLAG (**16E7**) plasmids in the presence of pcDNA-mycRb. (**D**) Immunoblots of Saos2 lysates cotransfected with either pcDNA3.1(−)-FLAG (**Vector**), pcDNA3.1(−)-5E7FLAG (**5E7**) or pcDNA3.1(−)-16E7FLAG (**16E7**) plasmids in the presence of pcDNA-mycRb and increasing concentrations of the proteasome inhibitor MG132. Lower panel shows the relative quantification of pRb with respect to eGFP. Note that MG132 inhibits pRb reduction mediated by 5E7 and 16E7. (**E**) Foreskin PHK cells were infected with retroviruses generated by pLZRS-E7-IRES-GFP vectors. Immunoblots prepared using specific antibodies showed reduced protein levels of pRb, p107 and p130 upon transduction with E7 from both HPV5 and 16. 5E7 was visualized with an anti-FLAG antibody, and 16E7 with an anti-16E7 antibody. E7 induced the expression of proliferation markers (PCNA and cyclin A), tumor suppressor p53, the proapoptotic effector Bax and the cell cycle inhibitor p21. No increase in the cell cycle inhibitor p16 was observed.

The transforming activities of the E7 oncogenes of HR-HPVs are mainly attributable to their ability to inhibit pocket proteins by reducing their protein levels. To confirm such activity in HPV5 E7 protein, cotransfection experiments were conducted using plasmids expressing E7-Flag and pRb genes in the pRb-deficient Saos2 human osteosarcoma cell line. Also, we cotransfected an eGFP-expressing plasmid as a control of transfection efficiency. Our results show a clear decrease in pRb protein levels upon transfection with HPV16 E7 (16E7) (Fig. 1A), as reported elsewhere [26]. Interestingly, the E7 protein from HPV5 shows a similar effect (Fig. 1A). As a negative control, E7 from HPV10 did not produce such a reduction, as already described [36]. Retinoblastoma protein reduction was more efficient when increasing amounts of the cotransfected E7-plasmids were used, both for HPV5 E7 and for HPV16 E7 (Fig. 1B). This confirms the reduction is dependent on E7-dose. The E7 effect on pRb levels could require direct binding. Using purified GST-E7-Flag proteins (Fig. S2A and B), we showed that E7 from HPV5 (as well as HPV10 and 16) is able to interact with pRb *in vitro* using lysates obtained from the human skin HaCaT cell line (Fig. S2C) or purified His-pRb (Fig. S2D), as has been described [22], [57], [58].

Mutational analyses indicate that the LxCxE motif of the N-terminal domain of HPV16 E7 protein is necessary for binding to pocket proteins. Using the Saos2 cotransfection system and E7 mutants (Materials and Methods), we confirmed that the capacity of the cutaneous E7 protein to reduce pRb levels by HPV5 E7 was dependent on an intact LxCxE motif, similarly to the HPV16 E7 protein (Fig. 1C), linking the binding ability with the effect on retinoblastoma protein levels.

HR-HPV E7 proteins decrease pRb levels by driving their degradation through the proteasome pathway. Using the Saos2 cotransfection system, we showed that steady-state pocket protein levels increase with MG132 concentrations, both for HPV5 E7 and HPV16 E7, indicating that HPV5 E7 might induce pRb destabilization through the proteasome, in a manner similar to HPV16 E7 (Fig. 1D).

Overall, these results indicate that expression of the HPV5 E7 protein induces pRb reduction, and that this reduction is at least in part mediated by proteasome-mediated degradation.

### E7-transduced Human Skin Grafts Display Stable Transgene Expression

In order to test the feasibility of our human skin grafting model for HPV analysis, we expressed the E7 genes from HPV5 and 16 by retroviral transduction of foreskin PHK cells to generate bioengineered skin, which was afterward transplanted onto the back of nude mice (see Materials and Methods). The added value of this approach is the grafting time, since the skin can be maintained for months on the animals. Thus, the long-term effect of HPV16 E7 expression in a properly assembled, mature human skin can now be analyzed *in vivo*. Moreover, the transgene expression from the retroviral construct may be easily monitored with eGFP as the E7 and eGFP genes are translated from the same mRNA transcript by the insertion of an IRES sequence between both open reading frames (see Materials and Methods).

Prior to grafting, PHKs infected with E7-coding retroviruses expressed the E7 and eGFP transcripts as demonstrated by qRT-PCR (Fig. S3A). The expression of E7 proteins in PHKs also promoted the reduction in endogenous pRb, p107 and p130 protein levels (Fig. 1E), but not in their corresponding transcripts, as determined by qRT-PCR (Fig. S4). However, the existence of residual pocket protein expression is explained because not all infected PHKs were successfully transduced (Materials and Methods, Fig. S1). In agreement with the reduction in pocket proteins, augmented expression of proliferation markers PCNA and cyclin A was detected. We also observed a slight increase in p53 expression, concomitant with the increase in the proapoptotic Bax protein and the cell cycle inhibitor p21, both directly regulated by p53. However, no increase was observed in p16, another cell cycle inhibitor normally overexpressed in HR-HPV-infected human biopsy specimens, suggesting that E7 alone might not deregulate p16 in human PHKs. In summary, PHKs transduced with HPV5 E7 share molecular features with oncogenic 16E7.

The E7-transduced PHK cells were used to generate bioengineered skin and subsequent grafting. Overall, 29 out of 36 transplants could be maintained until the end of the grafting time, indicating a high percentage of grafting success (80%) (Table 1) (Materials and Methods). EGFP expression was monitored by qRT-PCR (Fig. S3A), by green fluorescence visualized in the intact xenografts (Fig. S3B and C), and by immunostaining with a specific anti-eGFP antibody (Fig. S5). The patched immunostaining pattern of eGFP may be explained because grafting was performed with the pool of infected PHKs, containing both eGFP-positive (truly transduced) and negative cells. In all cases, E7 expression was also monitored by qRT-PCR (Fig. S3A). Overall, the transplants expressed the E7 genes at the time of collection, demonstrating that the humanized mouse model is able to unveil the long-term effects of HPV oncogenes.

**Table 1:** Histopathology of skin transplants.

Transplant Genotype	Graft ID	Grafting time (months)	General	Atrophy	Acanthosis	Hyperkeratosis	Papillomatosis	Suprabasal Mitosis	Nuclear atypia	Parakeratosis	Hypergranulosis	Focal apoptosis
Vector	1	3	normal									
	2	3	abnormal	yes		yes						
	3	3	normal									
	4	3	abnormal		yes							
	13	3	normal			mild				yes		
	14	3	normal			mild						
	15	3	normal									
	16	3	abnormal	yes		yes						
	25	6	normal									
	26	6	normal									
	27	6	abnormal							mild		yes
	28	6	abnormal	yes		yes						
5E7	5	3	normal									
	6	3	normal									
	17	3	normal									
	18	3	abnormal	yes		yes						
	19	3	normal		focal							yes
	20	3	abnormal	yes		yes						
	29	6	abnormal		focal	mild					yes	
	30	6	normal									
16E7	9	3	abnormal		yes	mild	yes	yes	yes		yes	yes
	11	3	abnormal		yes	yes	yes	yes	mild	yes	yes	
	12	3	abnormal		yes	yes	yes	yes	mild			yes
	21	3	abnormal		yes	yes		yes	yes			yes
	23	3	abnormal		yes	yes	yes	yes	yes		yes	
	33	6	abnormal		yes	yes	yes	yes	yes	yes	yes	yes
	34	6	abnormal		yes	yes	yes	yes	yes		yes	yes
	35	6	abnormal		yes	yes	yes	yes	yes	yes		yes
	36	6	abnormal		yes	yes	yes	yes			yes	yes

### E7-modified Engrafted Human Skin Shows Histological Features Compatible with HPV-associated Lesions

**Figure 2:**
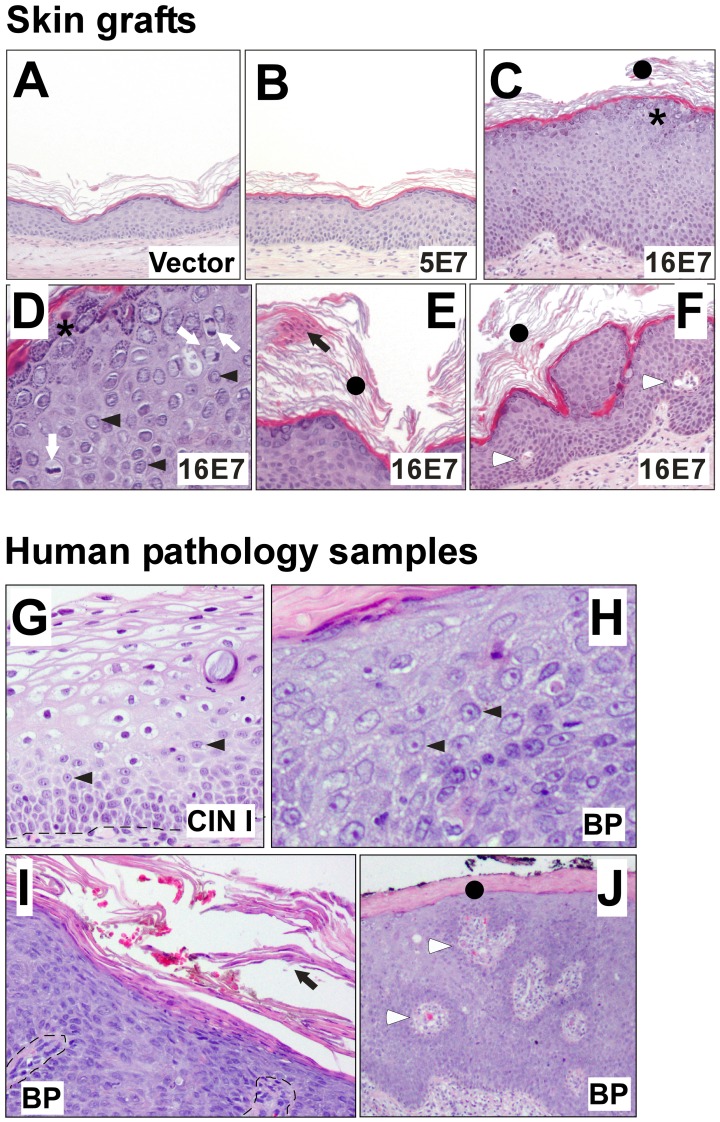
Histopathology of E7-grafts and human HPV-infected pathology samples. H&E staining of representative samples of control vector, HPV5 E7 and HPV16 E7 transplants [**Skin grafts:** upper images (**A** to **F**)], as well as CIN1 and bowenoid papulosis (BP) human HR-HPV-infected samples [**Human pathology samples**: lower images (**G** to **J**)]. The histology of vector and 5E7 samples is similar to that of normal skin, with basal, spinous, granulous and cornified layers properly assembled in a mature, differentiated squamous epidermal epithelium of normal thickness (**A** and **B**). All 16E7-grafts display epidermal acanthosis (**C**). Features observed in human HR-HPV-infected lesions are present in HPV16 E7-grafts including suprabasal mitosis (white arrows in **D**), nuclear atypia (black arrowheads in **D,** **G** and **H**), hyperkeratosis (black circles in **C**, **E**, **F**, and **J**), parakeratosis (black arrows in **E** and **I**), papillomatosis (**F**), hypergranulosis (asterisks in **C** and **D**) and capillaries (white arrowheads in **F** and **J**).

Through dermatoscopic visualization, macroscopic alterations could be identified in HPV16 E7-grafts as compared to controls. These alterations included epidermal thickening and desquamation, probably associated with hyperkeratosis (Fig. S6), which were not observed in HPV5-grafts. Moreover, HPV16 E7 samples also displayed papillomatous structures with capillaries and crypts (Fig. S6). These macroscopic features resemble those of human viral warts. Histological analysis of the transplants confirmed these observations, and showed that the HPV16 E7 gene induced phenotypic changes when compared with control grafts (Fig. 2 and Table 1). Most HPV16 E7 samples showed acanthosis associated with hyperplasia, suprabasal mitotic figures, hyperkeratosis, parakeratosis, hypergranulosis, nuclear atypia, papillomatosis and capillaries (Fig. 2C–F). No significant histopatological differences were observed between 5E7 and control vector grafts (Fig. 2A and B). Since similar features are present in cervical intraepithelial neoplasia (CIN) [59] (Fig. 2G), cervical carcinoma (CC), bowenoid papulosis (BP) lesions (Fig. 2H–J), vulvar intrapitelial neoplasia (VIN) or wart-like lesions [60], we demonstrated that 16E7-expressing, reconstituted human bioengineered skin transplanted onto nude mice, could constitute a faithful model of HPV-pathobiology.

### Epithelial Differentiation and Proliferation in Engrafted Skin

**Figure 3:**
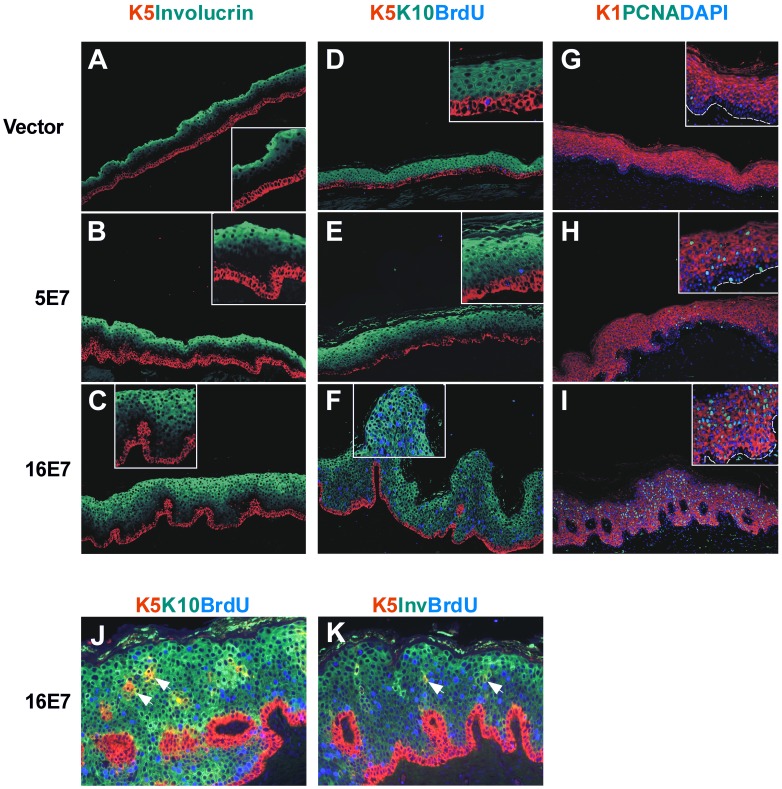
Epidermal differentiation and proliferation markers in E7-grafts. Sections of paraffin-embedded grafts were processed for immunofluorescence staining with antibodies to epithelial differentiation markers (basal cytokeratin 5, early suprabasal cytokeratin 1 and 10, and late suprabasal involucrin) and proliferation markers (BrdU, PCNA). As in normal skin, cytokeratin 5 (K5) is expressed in the basal layer, cytokeratin 10 (K10) and 1 (K1) in early suprabasal cells, and involucrin (Inv) in late differentiated cells in control vector samples (**A**, **D** and **G**). A similar phenotype was observed in the HPV5 E7 samples (**B**, **E** and **H**). An expansion of involucrin positive cells downwards was observed in HPV16 E7-transplants (**C** and **K**). BrdU and PCNA, normally present in some basal cells (**D** and **G**), are ectopically present in suprabasal cells of HPV16 E7 samples (**F** and **I**). Focal areas of suprabasal PCNA positive cells occur in HPV5 E7 samples (**H**). Importantly, suprabasal K5/K10 positive (**J**) or K5/involucrin positive cells (**K**) were observed in 16E7-grafts. Where indicated, DAPI staining was used to visualize cellular nuclei. **K5Inv**: staining with both K5 and involucrin antibodies; **K5K10BrdU**: staining with K5, K10 and BrdU antibodies; **K1PCNADAPI**: staining with K1, PCNA and DAPI. Dashed line indicates the location of the basal membrane.

To confirm our histology findings, suggestive of abnormal differentiation, we then explored the expression of epidermal differentiation markers by immunofluorescence. Cytokeratin K5, a marker of basal cells, showed a normal pattern in all the transplants (Fig. 3A–C, D–F), although K5-positive cells could eventually be seen in suprabasal layers in HPV16 E7-samples (Fig. 3J and K). Remarkably, these cells also exhibited the expression of cytokeratin K10 (early differentiation marker) (Fig. 3J) or involucrin (late differentiation marker) (Fig. 3K), revealing the known ability of HPV16 E7 expression to uncouple proliferation and differentiation processes. Normal suprabasal K10 expression was observed in all cases (Fig. 3D–F), but areas of reduced expression could be seen in HPV16 E7-transplants (Fig. 3F). Finally, involucrin expression was normally confined to the uppermost layers of spinous cells and the granulous layer in controls (Fig. 3A), whereas an expansion of involucrin expression was observed in the HPV16 E7-transplants (Fig. 3C), in agreement with the observed thickening of highly differentiated cell layers. No major changes were observed in HPV5 E7-transplants (Fig. 3B).

**Figure 4:**
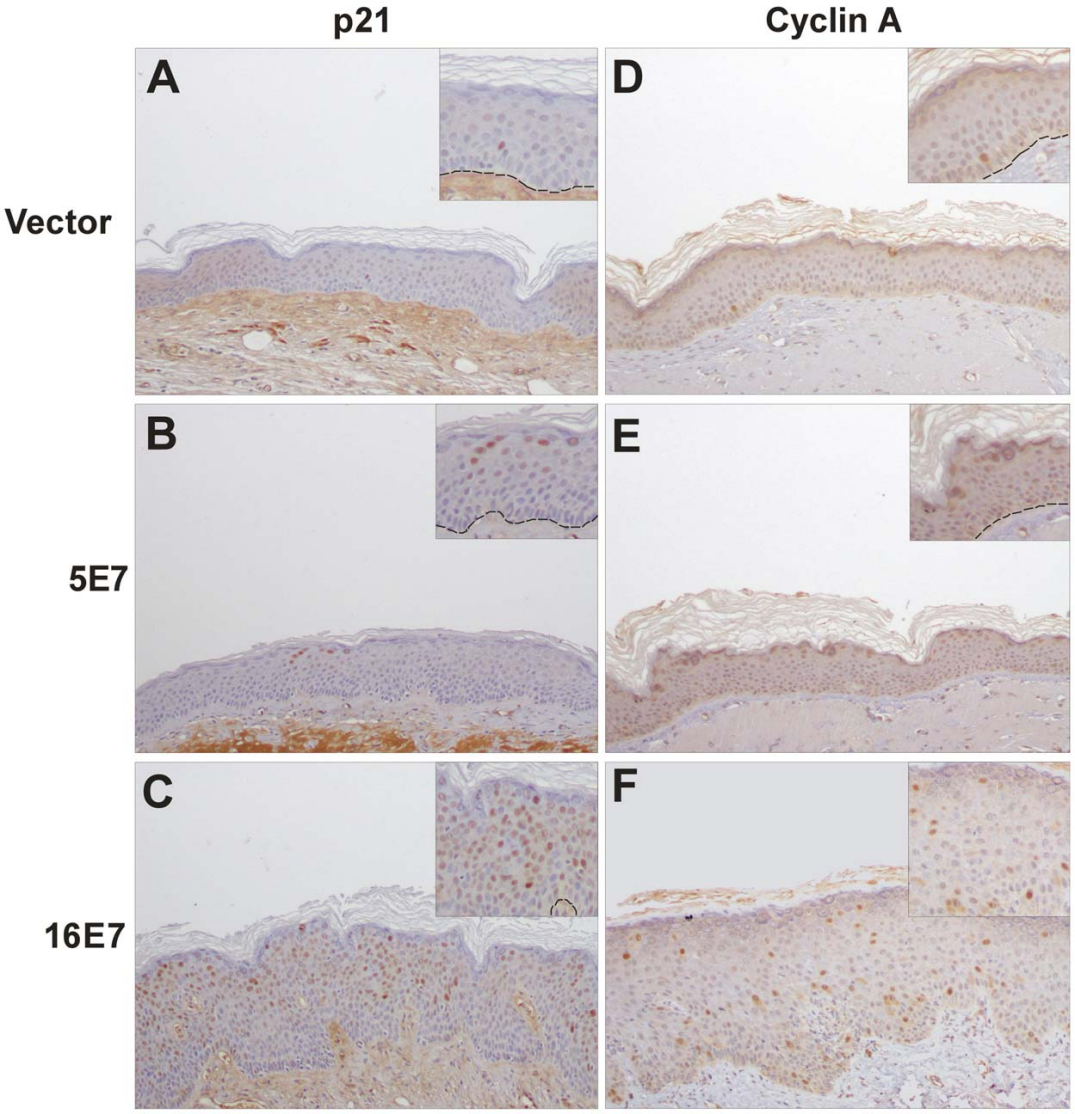
Induction of p21 and cyclin A in E7-grafts. Sections of paraffin-embedded grafts were processed for immunohistochemistry staining with antibodies to cell cycle inhibitor p21 and cell cycle regulator cyclin A. With respect to control vector grafts (**A** and **D**), both p21 and cyclin A proteins are induced in suprabasal cells of HPV16 E7 transplants (**C** and **F**) and in focal HPV5 E7 areas (**B** and **E**). In the inserts, dotted lines indicate the location of the basal membrane.

The hyperplasic phenotype observed in HPV16 E7 engrafted skin suggested augmented cell proliferation. This was further demonstrated by BrdU incorporation and PCNA expression in basal and suprabasal cells (Fig. 3F, I–K) compared to controls (Fig. 3D and G), that reached the upper layers of the grafted human skin. These findings support HPV16 E7 expression uncoupling proliferation and differentiation processes. In contrast, ectopic suprabasal proliferation was only detected in focal areas of the HPV5 E7-transplants (Fig. 3E and H). The HR-HPV E7 expression induces the expression of cell cycle regulators p21 and cyclin A in organotypic cultures and in pathological HPV-infected samples [61]–[63]. Through immunohistochemistry, we confirmed the virtually absent expression of both proteins in the bioengineered vector transplants (Fig. 4A and D). However, expression was focally observed in HPV5 E7-samples (Fig. 4B and E) or throughout HPV16 E7-grafts (Fig. 4C and F), being more evident for p21. The augmented expression of PCNA, p21 and cyclin A proteins was also observed in PHKs soon after retroviral infection, before transplantation (see above, Fig. 1E).

These results indicate that histological features reflecting increased proliferation (acanthosis or suprabasal mitosis) are associated with the induction of proliferation markers and cell cycle regulators in the case of 16E7. Grafts expressing E7 from HPV5 did not display evident histological changes (see above, Fig. 2 and Table 1), but deregulation of such proteins could be eventually observed. In line with this finding, the overexpression of p21 determined at the mRNA level was only significant in the16E7 transplants (Fig. S7), pointing to the incomplete activity of 5E7 in PHKs upon grafting.

### Apoptosis in E7-engrafted Skin

**Figure 5:**
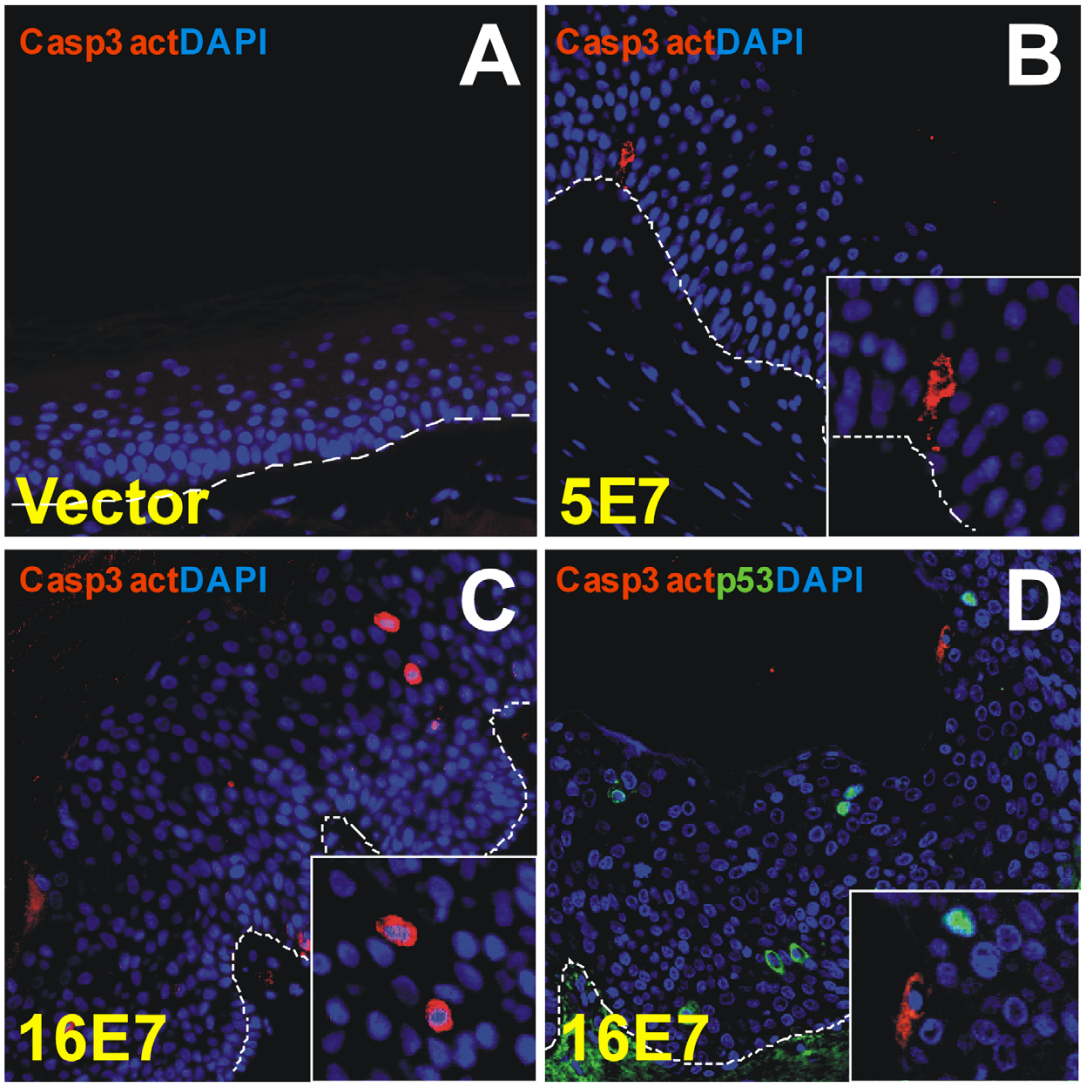
E7-grafts showed no significant apoptosis. Sections of paraffin-embedded grafts were processed for immunofluorescence staining with antibodies to the apoptosis markers caspase-3 active and p53. Caspase-3 active is not expressed in control vector samples (**A**), but some scattered positive cells are observed in HPV5 E7 (**B**) and HPV16 E7 grafts (**C** and **D**). No p53 staining was observed in control and HPV5 E7 samples (not shown), but eventual, p53-positive cells were observed in the HPV16 E7-transplants (**D**). Dotted line indicates the location of the basal membrane. Inserts in panels **B**–**D** show magnified areas of staining. DAPI was used to visualize cell nuclei.

The HPV16 E7 protein stabilizes the p53 tumor suppressor and induces apoptosis in cultured cells [24]. In agreement, we found that the E7-transduced PHKs prior to grafting show moderately increased expression of p53 and Bax, which is a proapoptotic inducer directly regulated by p53 (see above, Fig. 1E). However, we wanted to determine apoptosis in skin grafts using both p53 and active caspase-3, a processed protein which signals both extrinsic (death ligand) and intrinsic (mitochondrial) apoptotic pathways [64]. The results indicated no significant programmed cell death in control vector (Fig. 5A) or in the E7-transplants (Fig. 5B and C), although some few p53 or active caspase-3 positive cells appeared in HPV16 E7 grafts (Fig. 5D). In conclusion, E7 expression in the transplants appears to induce no substantial cell death. However, as caspase-3 is cleaved by HR-HPV-infection upon epithelial differentiation to induce viral genome amplification [65], we cannot discard that caspase-3 positive cells are non-apoptotic cells.

### Differential Activities of E7 Proteins from HPV5 and 16 in Skin Grafts

**Figure 6:**
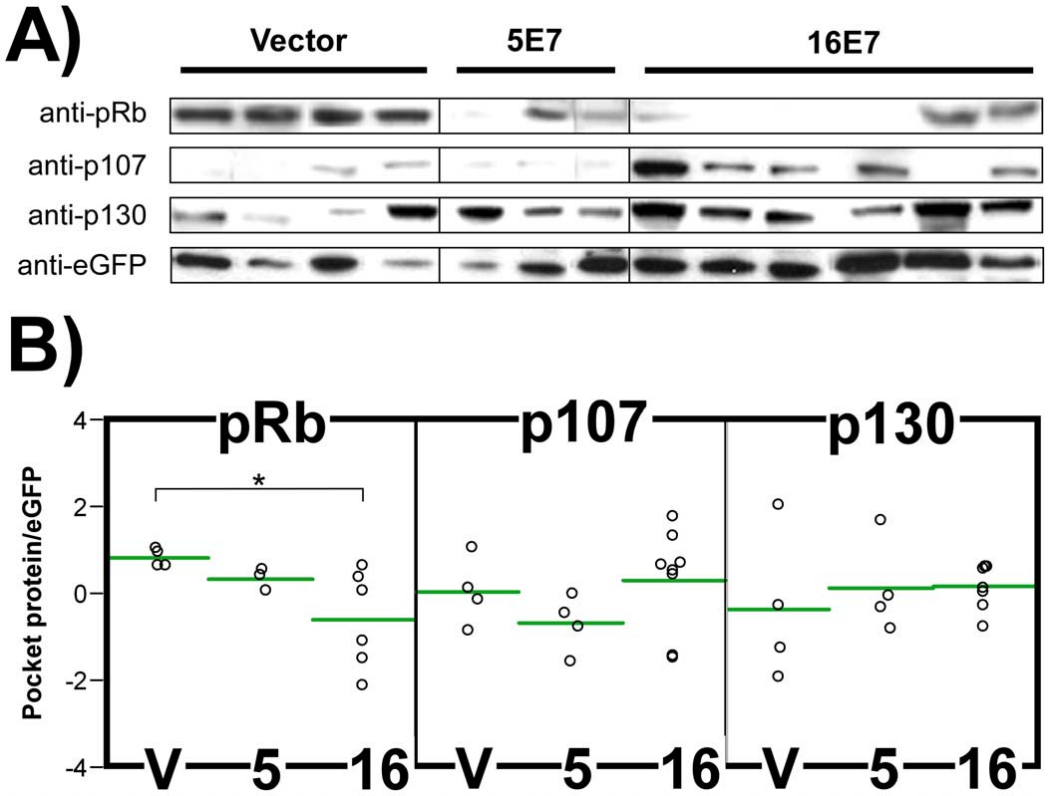
Pocket proteins expression in skin grafts. (**A**) Pocket protein and eGFP expression was analyzed by immunoblotting with specific antibodies using graft protein lysates from control vector, 5E7 and 16E7 samples. (**B**) Pocket protein and eGFP protein expression bands were quantified and relative (pocket protein/eGFP) values plotted. Horizontal green lines represents mean relative values. According to Student’s t-test, significant differences were only detected in pRb/eGFP expression values between vector and 16E7 samples (threshold p-val<0.05). *: p-val<0.05.

Histological and molecular characterization of the skin grafts demonstrated differences between the phenotypic effects of the expression of cutaneous HPV5 E7 and mucosal HPV16 E7, despite the similarities observed *in vitro* using transfected Saos2 cells and transduced foreskin PHKs in culture (see above, Fig. 1). Immunohistochemical markers of proliferation and cell cycle regulators revealed an ectopic suprabasal expression in patches of the 5E7-grafts, while the effect was mostly general in the 16E7-gratfs. A possible explanation is that the LTR regulatory sequences might be silenced by epigenetic mechanisms in the case of 5E7-grafts. Detection of 5E7 protein expression by immunohistochemistry was not possible as the anti-Flag antibodies produced no specific signal. However, i) qRT-PCR of 5E7 and eGFP genes, ii) green fluorescence visualization of intact xenografts, and iii) eGFP immunohistochemistry (Fig. S3 and S5) indicated active transgene expression. This findings, together with absence of apoptosis (see above, Fig. 5), discarded a negative selection of HPV5 E7-transduced cells during grafting time. An alternative explanation is that HPV5 E7 cannot target pocket proteins with a similar efficiency as that observed *in vitro*. We tested this hypothesis by obtaining protein lysates from the skin grafts, and analyzing the expression of the pocket proteins (Fig. 6A). We also determined their expression relative to eGFP, which is translated from the same transcript as E7 in order to normalize differences due to variability between grafts (Fig. 6B). Interestingly, results showed that pRb expression is significantly reduced in 16E7-transplants, in agreement with the increased expression of proliferation markers. However, pRb levels were not significantly modified in skin grafts with the HPV5 E7 gene. No significant differences were observed in p107 and p130 expression for both 5E7 and 16E7 samples. In view of these results, we suggest that the absence of a phenotype for the cutaneous E7 gene might be the lack of effective pRb reduction that occurred *in vivo*.

### Validation of the E7-grafts as a Model for HPV-associated Disease

**Figure 7:**
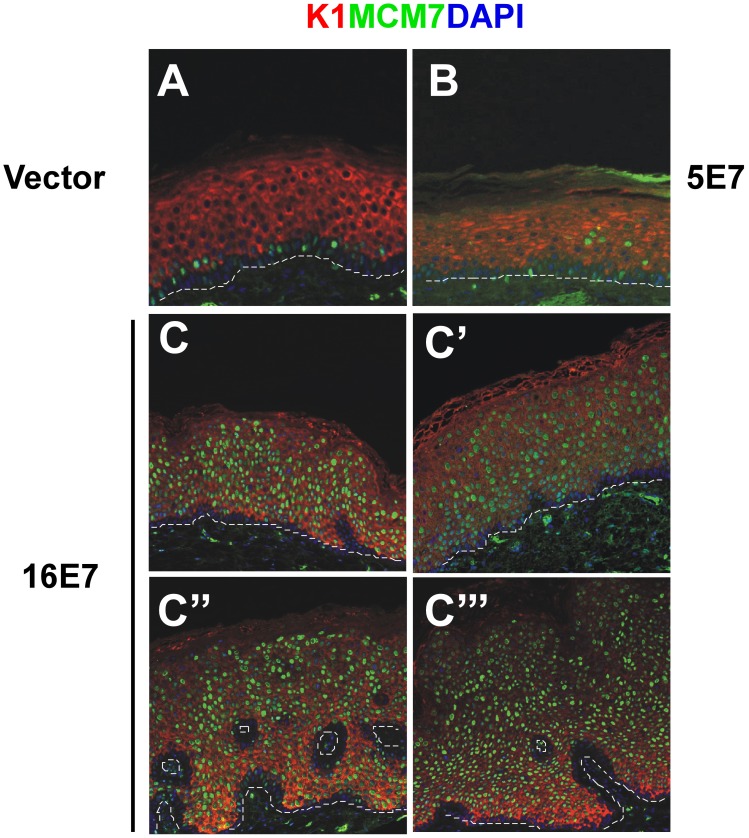
HPV surrogate marker MCM7 is ectopically expressed in E7-transplants. Sections of paraffin-embedded grafts were processed for immunofluorescence staining with antibodies to MCM7. MCM7 is normally confined to the basal layer as in control vector transplants (**A**). Suprabasal expression is eventually observed in HPV5 E7-samples (**B**). Importantly, MCM7 is expressed in HPV16 E7-grafts in most suprabasal cells, reaching the uppermost spinous cells. Representative images of different 16E7-grafts are shown (**C, C’, C’’, C’’’**).

To validate our humanized mouse model of HPV-pathology analyses, we examined the expression of several biomarkers previously identified as surrogate markers of HPV infection in pathology samples. The cellular protein MCM7, a helicase involved in DNA replication, is induced by HPV-E7 expression in clinical samples of CIN and carcinoma lesions [66]. Immunohistochemistry indicated MCM7 expression in basal cells of control vector samples (as expected) (Fig. 7A). Ectopic suprabasal expression was focally observed in HPV5 E7 grafts (Fig. 7B) (as for PCNA, p21 and cyclin A, see above Fig. 3 and 4). In contrast, MCM7 displayed continuous ectopic suprabasal expression in HPV16 samples that reached the uppermost spinous cells (Fig. 7C–C’’’).

**Figure 8:**
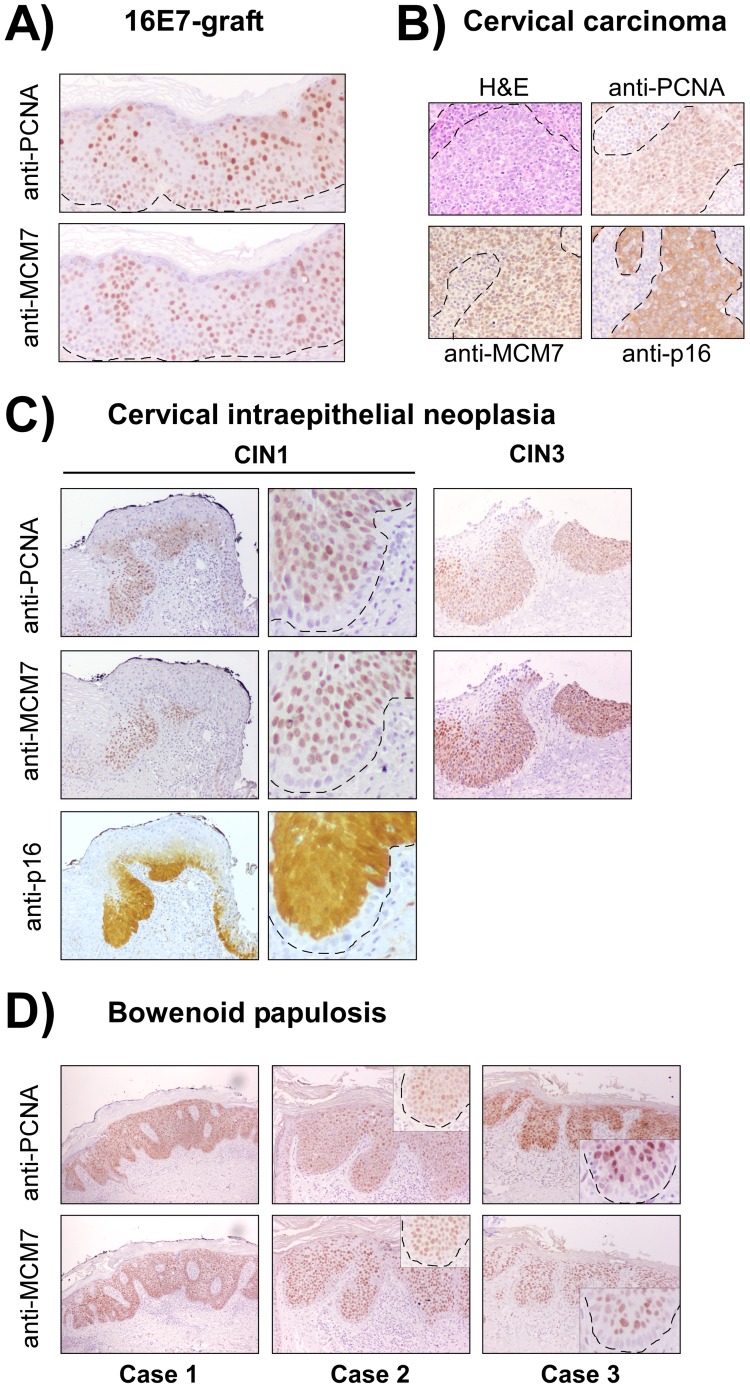
Similarities between 16E7-grafts and HPV-infected anogenital neoplasias identified by immunohistochemistry. (**A**) Both PCNA and MCM7 displayed similar ectopic expression in most suprabasal cells of 16E7-grafts. In the basal layer, areas of expression coexisted with expression-free areas. (**B**) The same patterns were observed in tumor areas of cervical carcinoma samples where p16 was also present. PCNA and MCM7 expression expands progressively upwards in CIN1 and CIN3 (**C**), while most basal and subrabasal cells were stained in different cases of bowenoid papulosis (**D**). Middle panels in **C** and inserts in **D** highlight areas of basal cells showing negative staining for MCM7, PCNA and p16 (the latter, in CIN1). Dotted lines indicate the location of the basal membrane.

**Figure 9:**
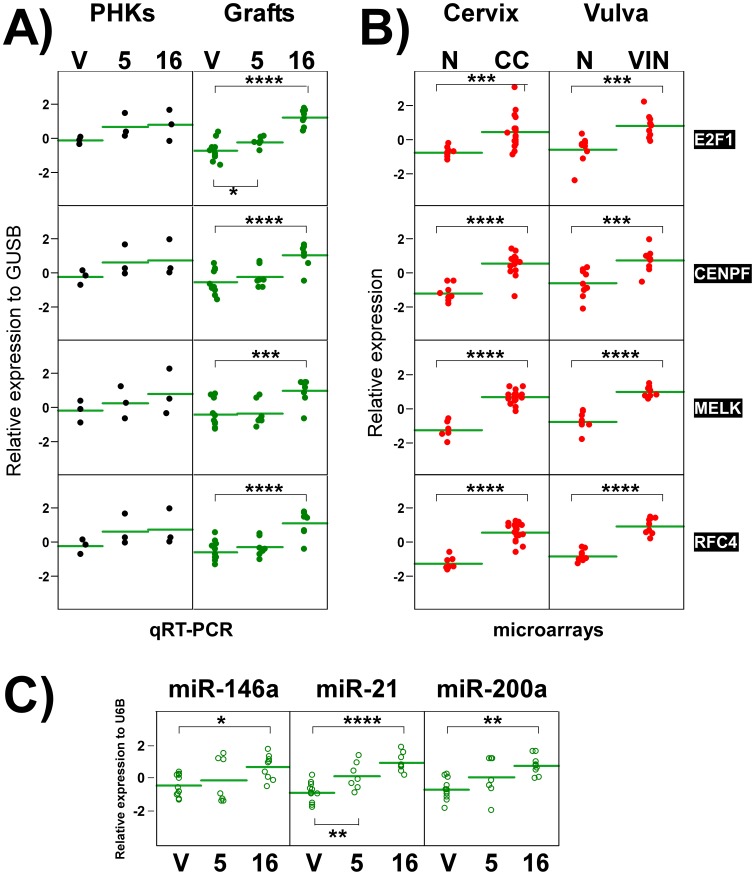
RNA expression quantification of oncogenic HPV biomarker genes and miRNAs. mRNA quantification of E2F1, CENPF, MELK and RFC4 genes was conducted by qRT-PCR using RNA purified from PHK cells or grafts, or obtained from reported microarray experiments for CC or VIN. Each dot represents an individual sample. Horizontal lines represent mean values in each sample group. (**A**) All genes were induced in E7-transduced PHKs, though not significantly (Student’s t-test threshold p-val<0.05). Significant overexpression was also observed for all genes in HPV16 E7-transplants, but only for E2F1 in HPV5 E7. Shown are log_2_-based, z-values of expression relative to housekeeping GUSB in PHK cells and transplants (Materials and Methods). (**B**) Microarray expression patterns of each gene in CC and VIN infected with HR-HPVs are shown as log_2_-based, z-values (Materials and Methods). All genes display significant overexpression in CC with respect to cervix uteri (N), and in VIN with respect to normal vulva (N). Horizontal lines represent mean values in each sample group. *: p-val <0.05, **: p-val <0.005, ***: p-val <0.0005, ****: p-val <0.00005. **V**: control vector; **5**: HPV5 E7; **16**: HPV16 E7. (**C**) miRNA was quantified by qRT-PCR for miR-146a, miR-21 and miR-200a, using RNA purified from skin grafts. Shown are log_2_-based, z-values of expression relative to housekeeping U6B. Each dot represents an individual sample value. All miRNAs were overexpressed in HPV16 E7-grafts. Only miR-21 displayed significant deregulation in transplants expressing HPV5 E7. Horizontal lines represent mean values in each sample group. *: p-val <0.05, **: p-val <0.005, ***: p-val <0.0005, ****: p-val <0.00005. **V**: control vector; **5**: HPV5 E7; **16**: HPV16 E7.

Further, 16E7-grafts and clinical samples of mucosal HR-HPV-associated lesions (CIN, CC and BP) were compared by immunohistochemistry, both for MCM7 and PCNA. Results revealed similar staining patterns for both proteins in the 16E7-grafts (Fig. 8A) and all the pathological cases (Fig. 8B–D, Fig. S8B and C). PCNA and MCM7 were ectopically expressed in the lower epithelial suprabasal layers of CIN1, and their expression expanded upwards in CIN3 (Fig. 8C, Fig. S8B), reaching almost all tumor cells in CC samples (Fig. 8B), as described elsewhere. Similar p16 staining patterns were produced in the CC or CIN samples (Fig. 8B and C). However, we could not observe differences in p16 expression between control and E7-grafts, by immunohistochemistry on skin grafts (Fig. S8A) or by immunoblots in PHKs (see above, Fig. 1E). Importantly, PCNA and MCM7 show a strong pattern similarity with BP human samples, where both proteins are expressed in most suprabasal cells (Fig. 8D, Fig. S8C). Moreover, close inspection showed that not all basal cells exhibited MCM7, PCNA or p16 expression in CIN1 (Fig. 8C) or BP (inserts in Fig. 9D), as occurs in 16E7 grafts (Fig. 7C–C’’’ and Fig. 8A). However, CIN3, CC and high grade BP did express both markers in all basal cells (Fig. 8B and C, Fig. S8B and C), pointing to a clonal selection of malignant infected cells in the basal layer during tumor progression.

We recently reported a meta-analysis of expression profiling experiments performed on HR-HPV infected human samples [67]. The overexpression was described of genes involved in DNA replication and proliferation such as *E2F transcription factor 1* (E2F1), *centromere protein F* (CENPF), *maternal embryonic leucine zipper kinase* (MELK) and *replication factor C (activator 1) 4* (RFC4) in various independent microarray studies performed on HPV-infected CC and head and neck squamous cell carcinomas (HNSCC). The expression of these genes was slightly induced in PHKs expressing E7 genes prior to grafting (Fig. 9A). In contrast, E2F1 is significantly overexpressed by both E7 genes in grafts (Fig. 9A). Furthermore, CENPF, MELK and RFC4 genes are overexpressed in HPV16 E7 expressing grafts (Fig. 9A). The expression patterns of these 4 genes in HR-HPV-infected VIN [53] and CC samples [52] (compared to their respective normal control tissues) are provided to demonstrate their similar deregulation trends (Fig. 9B).

MicroRNAs (miRNAs) are endogenous small non-coding RNAs capable of modulating gene expression by post transcriptional mechanisms. miRNAs are involved in numerous of normal or pathological cell processes, such as cancer. In HPV-infected CC or HNSCC, miR-145, miR-146a, miR-148a, miR-200a, miR-203 and miR-21 (among others) are deregulated, some of them in association with clinical variables [10], [68]–[71]. Thus, through qRT-PCR we examined the expression of these specific miRNAs in grafts. No expression differences were observed in the expression of miR-148a, miR-145 or miR-203 when compared to control vector grafts (Fig. S9). However, we found significant overexpression of miR-146a and miR-200a in HPV16 E7-grafts, and miR-21 in both HPV5 E7- and HPV16 E7-grafts (Fig. 9C).

Collectively, these observations indicate that the 5E7-grafts, despite the similarity with normal vector skin at the histology level, display a slight deregulation of HPV-biomarkers. In addition, 16E7-transplants exhibits molecular deregulation of HPV-infected carcinomas and support the idea that our model is able to recapitulates molecular the features that characterize HPV-associated pathologies.

## Discussion

The findings of our study indicate that HPV5 E7 is able to reduce pRb levels *in vitro*, in a manner that is most likely dependent on protein destabilization. Also, we adapted our previously described humanized murine model to analyze the long-term *in vivo* phenotypic consequences of E7 (from HPV5 and HPV16) expression. To validate the model, the functionality of transduced 16E7 was supported by expected E7 protein activity and alterations in epithelial homeostasis, histopathology, and biomarkers of HPV-associated neoplasia. The model emerges as a valid tool for dissecting the molecular pathogenesis of HPV as well as for pre-clinical testing of therapies for HPV-associated malignancy.

A major feature of our humanized model is the long-term stability of the transgene expression. Two sets of transplants were maintained for up to 3 months after engrafting, and one set was kept longer, for 6 months. No significant differences were observed between both transplant durations in terms of phenotype aggravation, transgene (E7 and eGFP) expression/silencing, or biomarker induction. Thus, unlike the situation with (non-engrafted) organotypic skin, pathogenesis could be analyzed during prolonged time periods (minimum of 6 months), which may allow for a more physiological analysis of E7 functions, and more adequate tests of toxicity and efficiency for new possible therapies. Although transgenic mice expressing HPV oncogenes represent good organism model systems, they might not fully recapitulate the human histopathology and molecular interactions due to possible intrinsic differences between humans and mice. Compared with transgenic mice, in the humanized model the consequences of E7 expression could be analyzed using human primary keratinocytes, as the true targets of HPV infection. Although the absence of a normal immunological system in *nu/nu* mice could be considered a disadvantage of the model, is worth considering that immunosuppression is likely to contribute to the malignant development of HPV-associated lesions. It is true, however, that this model is not useful to address antitumor therapies based on immune system interactions or induction.

The HPV16 E7 grafts established here showed histopathological and/or molecular features that can be observed in HPV-associated BP, VIN, wart-like lesions, CIN or CC. Of note, the augmented proliferation of basal and suprabasal cells, the disorganized differentiation, and the ectopic expression of p21 and cyclin A, are in line with previous findings in organotypic cultures and HPV-based transgenic mice, thus validating our model. In human clinical samples, immunostaining revealed that MCM7 and PCNA are restricted to the basal and/or early suprabasal cells in low grade CIN but are also overexpressed in the upper layers of the epithelia in high-grade CIN lesions, CC, and BP. Remarkably, grafts obtained upon expression of HPV16 E7 were found to express MCM7 and PCNA in all suprabasal layers up to the spinous cells of the skin epidermis, thus resembling high grade CIN lesions, CC and BP. However, patched expression in basal cells was observed in 16E7-grafts (Figures 7 and 8A), which may be a consequence of the coexistence of transduced and non-transduced cells. As basal cells expressing E7 displayed proliferative potential over E7-negative cells, they expanded upwards more efficiently while expressing MCM7 and PCNA in differentiated cells. Importantly, similar findings were observed in CIN1 and bowenoid papulosis samples, where positive and negative basal cells coexisted in the lesions (Fig. 8C and D).

The absence of p16 expression in 16E7-grafts is the major difference with HR-HPV-infected human pathological samples. Normally, p16 is expressed in CIN, CC and BP associated with the infection of oncogenic mucosal HPVs (mostly HPV16 and 18). Our results suggest that additional oncogenic events might be needed to induce ectopic expression of p16 in human lesions, such as co-expression with other viral genes (such as E6) and/or mutational events within the human genome. Further analysis must be performed to determine the molecular mechanisms by which p16 is induced HR-HPV-infected human samples.

Our biomarker characterization of the grafts revealed the induction genes such as E2F1, CENPF, MELK and RFC4, or miRNAs such as miR-21, miR-146a and miR-200a. Importantly, miR-21, which displays oncogenic and metastatic activity [72], is also overexpressed in cell lines and clinical samples of HNSCC [12] and CC [68], [69] and is associated with a poorer prognosis [73]. Although the possible mechanism of miR-21 induction by E7 in grafts is not known, the model can be used to analyze the *in vivo* consequences of miR-21 inhibition by pharmacological or genetic approaches. miR-146a expression is regulated by NF-κB signaling [74] and, accordingly, changes in miR-146a expression have been described not only in CC and other cancers [68], [69], but also in psoriasis and in skin inflammatory processes [75]. Consequently, the upregulation of miR-146a could potentially inhibit the activation of inflammation upon *in vivo* infection, thus contributing to the progression of asymptomatic HPV-associated pathology. Finally, we detected the overexpression of miR-200a in the HPV16 E7-grafts. Since miR-200a negatively regulates epithelial to mesenchymal transition [7], [76], its increased expression is in line with the lack of invasive properties of the HPV16E7-grafts.

The histology and molecular differences between 5E7 and 16E7-grafts could be partially explained by ineffective degradation of 5E7 over pRb. We are tempting to speculate that human skin SCC infected with HPV5 might need additional cellular mutations that could cooperate with virus-induced transformation, such as those produce by UV irradiation. Further experiments using UV irradiation may corroborate such hypothesis. In this sense, K14-HPV38 transgenic mice develop tumors upon chemical or UV stimuli [8]. However, we cannot discard that the cutaneous virus protein lacks other molecular activities that the HR-HPV16 E7 protein displays, such as inhibition of p21 function [77]–[79]. Whether p21 is active or not in the HPV5 E7-grafts remain to be ascertained, although protein expression was only observed in patched areas.

In summary, we present a new model system to analyze the functions of the HPV oncogenes in human keratinocytes that can be maintained for several months. This model represents a new *in vivo* tool, which could be combined with analyses in monolayer cell culture and organ systems for preclinical validation of therapies against HPV-diseases. The validity of the model is supported by our histopathological findings and the expression of several biomarkers of HPV infection and carcinogenesis. Additional experiments analyzing the cooperative roles of both E6 and E7 proteins merits further investigation, as both oncogenes are expressed in human tumor samples. We propose this model may be useful for preclinical tests designed to search out new efficient molecularly targeted therapies against HPV-associated lesions.

## Supporting Information

Figure S1 **Infection efficiency of E7-containing retroviral vectors in foreskin PHKs.** **A**) A representative example of a flow cytometer analysis is shown in which the percentage of PHK cells is plotted against eGFP fluorescence. **B**) Table shows the percentage of eGFP positive PHK cells per genotype (control vector, 5E7 or 16E7) for each infection set.
(TIF)

Figure S2 **GST-E7 pull-down experiments.** **A**) Immunoblots of purified GST and GST-E7Flag fusion proteins using an anti-FLAG antibody. **B**) Ponceau red staining of GST fusion proteins used as input for *in vitro* binding assays. **C**) GST fusion proteins were immobilized and incubated with total protein extracts of human HaCaT keratinocytes. Immunoblotting with pRb and GST specific antibodies showed that E7 proteins from HPV10, HPV5 and HPV16 interact with pRb. One tenth of the total cell extract used in the GST pull-down assay (input) was also analyzed. **D**) GST fusion proteins were immobilized and incubated with purified His-pRb. Immunoblotting with pRb, His and GST specific antibodies revealed that E7 proteins from HPV10, HPV5 and HPV16 interact with purified pRb.
(TIF)

Figure S3 **Stable transgene expression of E7 and eGFP genes in PHKs and skin grafts. A**) qRT-PCR analysis of eGFP, 5E7 and 16E7 genes in transduced PHKs before grafting or after the transplantation time. Shown are log_2_-based, z-values of expression relative to housekeeping GUSB in PHK cells and transplants (Materials and Methods). Horizontal lines represent means for each sample group. **V**: control vector; **5**: 5E7; **16**: 16E7. Normal macroscopic (**B**) or green fluorescence visualization (**C**) in grafts is due to the expression of the eGFP transgene.
(TIF)

Figure S4 **Pocket protein mRNA quantified in foreskin PHKs.** qRT-PCR was conducted for pRb, p107 and p130 genes obtained from retrovirally transduced PHKs. Shown are log_2_-based, z-values of expression relative to housekeeping GUSB in PHK cells and transplants (Materials and Methods). Each dot represents an individual sample. Horizontal lines represent means for each sample group. No reduction in mRNA levels of retinoblastoma family genes was observed upon E7 expression. **V**: control vector; **5**: 5E7; **16**: 16E7. No significant gene expression differences were detected between the samples as assessed by a Student’s t-test (threshold p-val<0.05).
(TIF)

Figure S5 **Immunostaining with anti-eGFP.** Patchy expression was observed in the grafts corresponding to the control vector, 5E7 and 16E7 samples. H&E staining of similar areas is also shown.
(TIF)

Figure S6 **Dermatoscopic images of human skin E7-grafts.** Representative images of control vector, 5E7, and 16E7 samples. Lower, right panel of 16E7 represents the highlighted area in the left 16E7 image. Similar features to human viral warts included: i) hyperkeratosis and papillomatosis (areas within dashed lines), ii) capillaries (black arrows, and upper insert) and iii) crypts associated with papillomatosis (white arrows and lower insert).
(TIF)

Figure S7 **Quantification of p21 mRNA expression.** qRT-PCR was conducted on p21, showing a moderate increase in PHK cells before grafting that was significantly augmented after transplantation in the case of HPV16 E7. Each dot represents an individual sample. Horizontal lines represent means for each sample group. Shown are log_2_-based, z-values of expression relative to housekeeping GUSB in PHK cells and transplants (Materials and Methods). **V**: control vector; **5**: 5E7; **16**: 16E7. A Student’s t-test detected significant differences in gene expression between the different samples (threshold p-val<0.05). ****: p-val<0.00005.
(TIF)

Figure S8 **HPV biomarker analysis in human infected anogenital neoplasias.** **A**) Immunohistochemistry of p16 using the CINtec Histology Kit on skin E7-grafts and cervical carcinoma samples. No differences were observed in transplants upon E7 expression. As expected, a CC sample was stained with the p16 antibody. PCNA and MCM7 expression patterns in clinical samples of cervical intraepithelial neoplasia (**B**) and high grade bowenoid papulosis (**C**). Dashed lines in **C** represent the basal membrane.
(TIF)

Figure S9 **qRT-PCR for miR-148a, miR-145 and miR-203 miRNAs in E7-transplants.** Each dot represents an individual sample. Horizontal lines represent means for each sample group. Shown are log_2_-based, z-values of expression relative to housekeeping U6B (Materials and Methods). **V**: control vector; **5**: 5E7; **16**: 16E7. A Student’s t-test was performed to detect significant differences in gene expression between the different samples (threshold p-val<0.05). No significant deregulation was observed.
(TIF)

Table S1 **Oligonucleotides used.**
(XLS)

Table S2 **Antibodies used.**
(XLS)
