# Supplementary figures and images for: A Humanized Mouse Model of HPV-Associated Pathology Driven by E7 Expression
Source: PLoS One. 2012 Jul 23;7(7):e41743. doi: 10.1371/journal.pone.0041743 (PMC3402432; doi:10.1371/journal.pone.0041743)

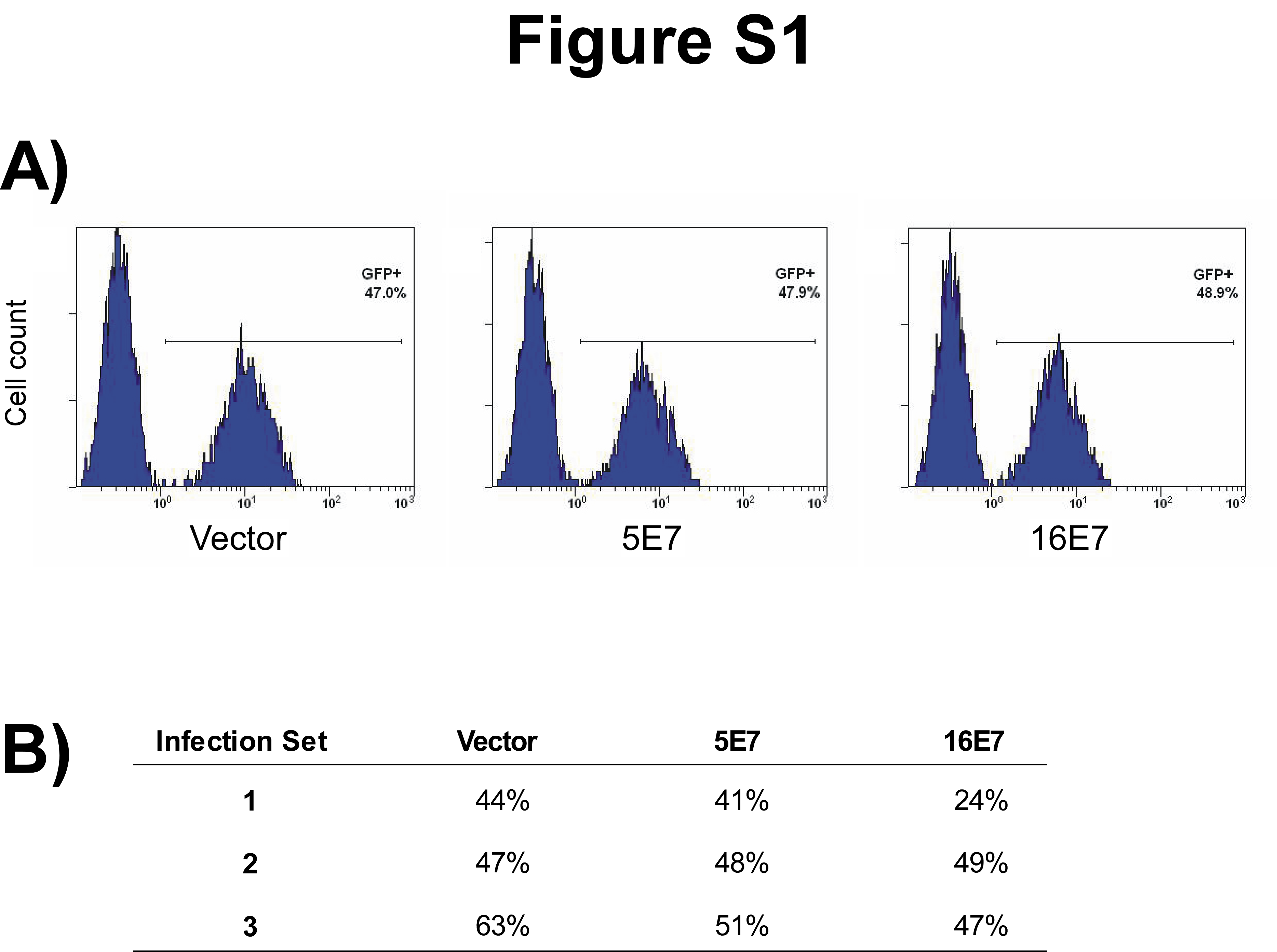

Supplement: Figure S1 — Infection efficiency of E7-containing retroviral vectors in foreskin PHKs. A) A representative example of a flow cytometer analysis is shown in which the percentage of PHK cells is plotted against eGFP fluorescence. B) Table shows the percentage of eGFP positive PHK cells per genotype (control vector, 5E7 or 16E7) for each infection set. (TIF) [file pone.0041743.s001.tif]

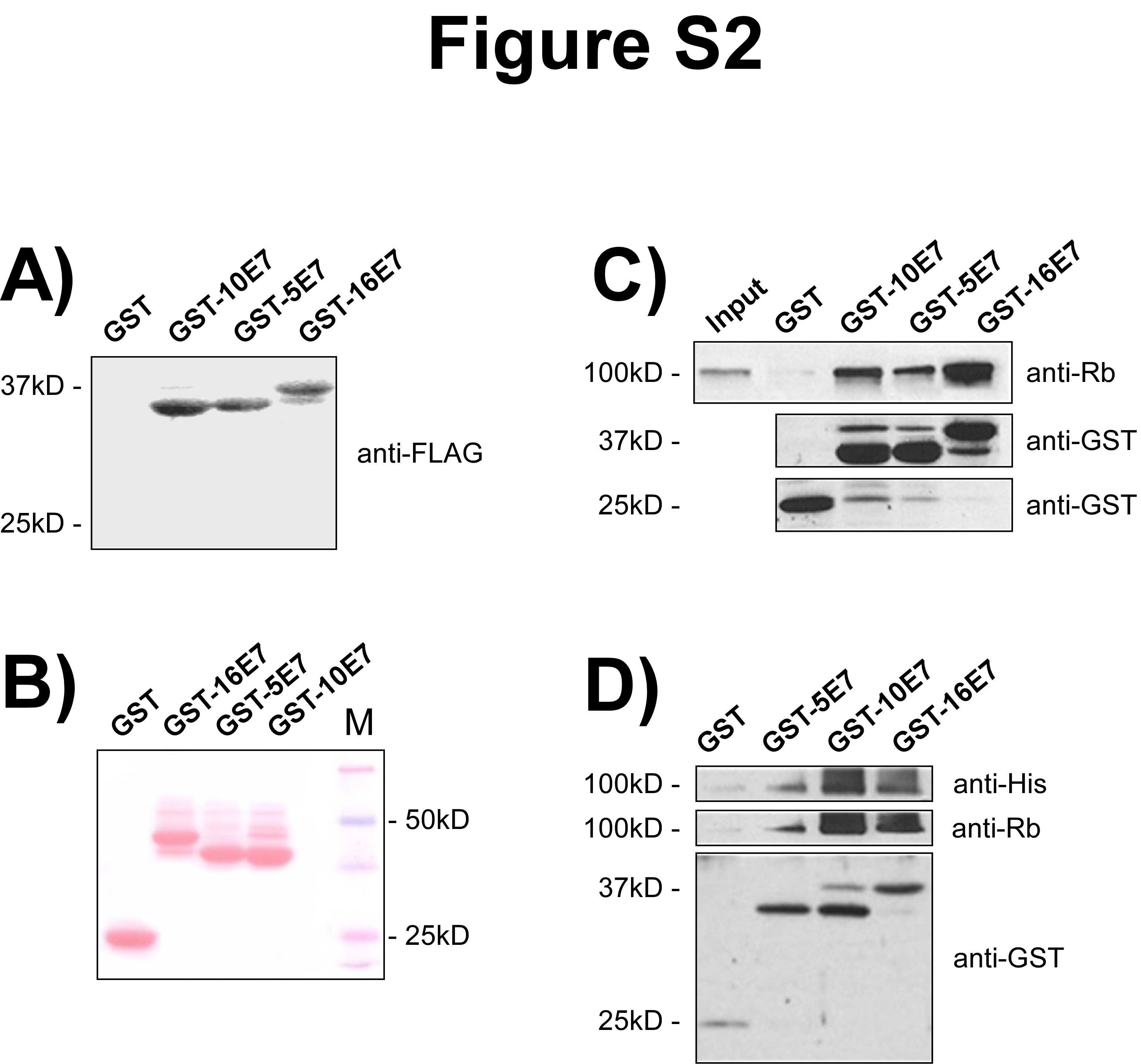

Supplement: Figure S2 — GST-E7 pull-down experiments. A) Immunoblots of purified GST and GST-E7Flag fusion proteins using an anti-FLAG antibody. B) Ponceau red staining of GST fusion proteins used as input for in vitro binding assays. C) GST fusion proteins were immobilized and incubated with total protein extracts of human HaCaT keratinocytes. Immunoblotting with pRb and GST specific antibodies showed that E7 proteins from HPV10, HPV5 and HPV16 interact with pRb. One tenth of the total cell extract used in the GST pull-down assay (input) was also analyzed. D) GST fusion proteins were immobilized and incubated with purified His-pRb. Immunoblotting with pRb, His and GST specific antibodies revealed that E7 proteins from HPV10, HPV5 and HPV16 interact with purified pRb. (TIF) [file pone.0041743.s002.tif]

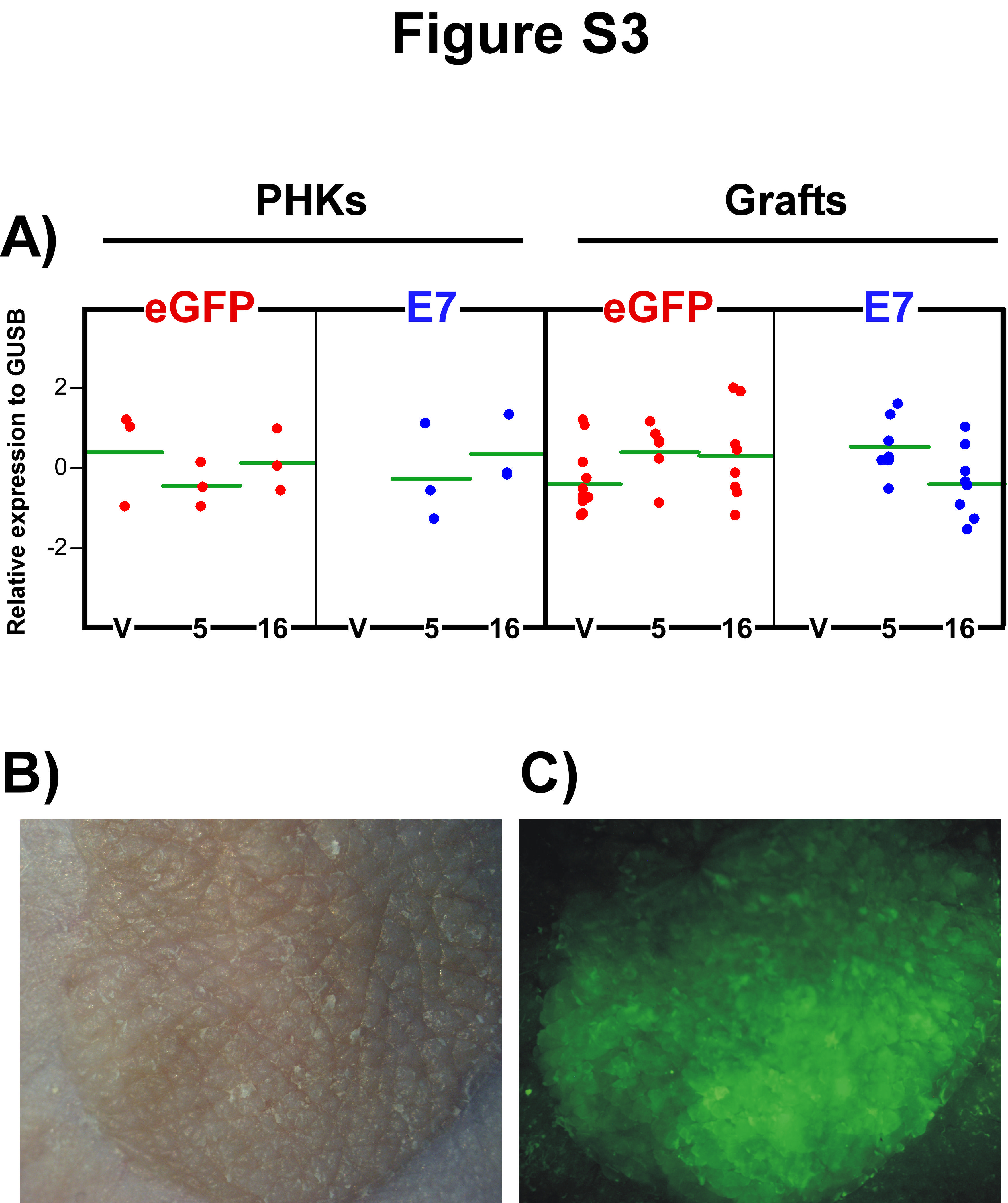

Supplement: Figure S3 — Stable transgene expression of E7 and eGFP genes in PHKs and skin grafts. A) qRT-PCR analysis of eGFP, 5E7 and 16E7 genes in transduced PHKs before grafting or after the transplantation time. Shown are log2-based, z-values of expression relative to housekeeping GUSB in PHK cells and transplants (Materials and Methods). Horizontal lines represent means for each sample group. V: control vector; 5: 5E7; 16: 16E7. Normal macroscopic (B) or green fluorescence visualization (C) in grafts is due to the expression of the eGFP transgene. (TIF) [file pone.0041743.s003.tif]

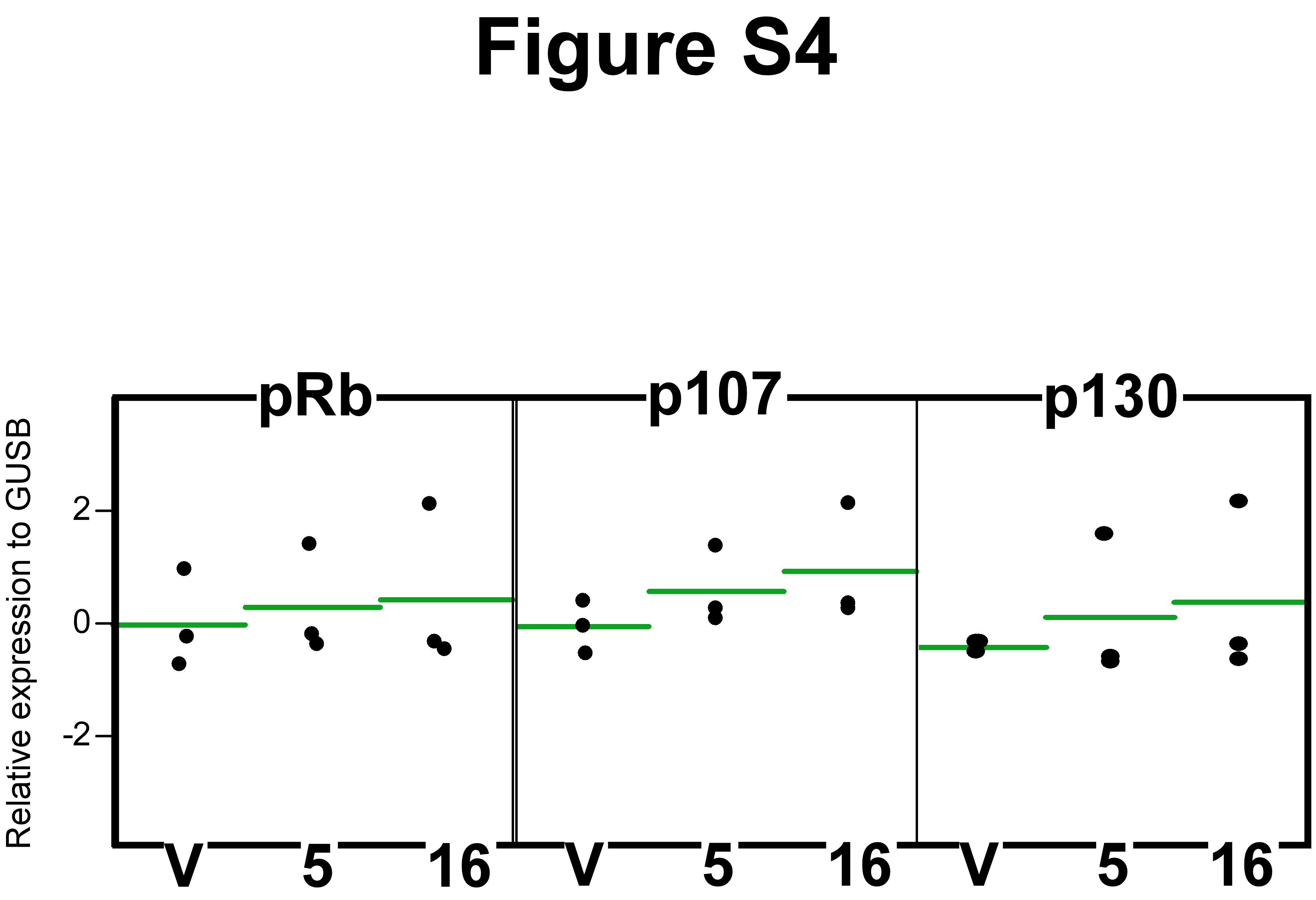

Supplement: Figure S4 — Pocket protein mRNA quantified in foreskin PHKs. qRT-PCR was conducted for pRb, p107 and p130 genes obtained from retrovirally transduced PHKs. Shown are log2-based, z-values of expression relative to housekeeping GUSB in PHK cells and transplants (Materials and Methods). Each dot represents an individual sample. Horizontal lines represent means for each sample group. No reduction in mRNA levels of retinoblastoma family genes was observed upon E7 expression. V: control vector; 5: 5E7; 16: 16E7. No significant gene expression differences were detected between the samples as assessed by a Student’s t-test (threshold p-val<0.05). (TIF) [file pone.0041743.s004.tif]

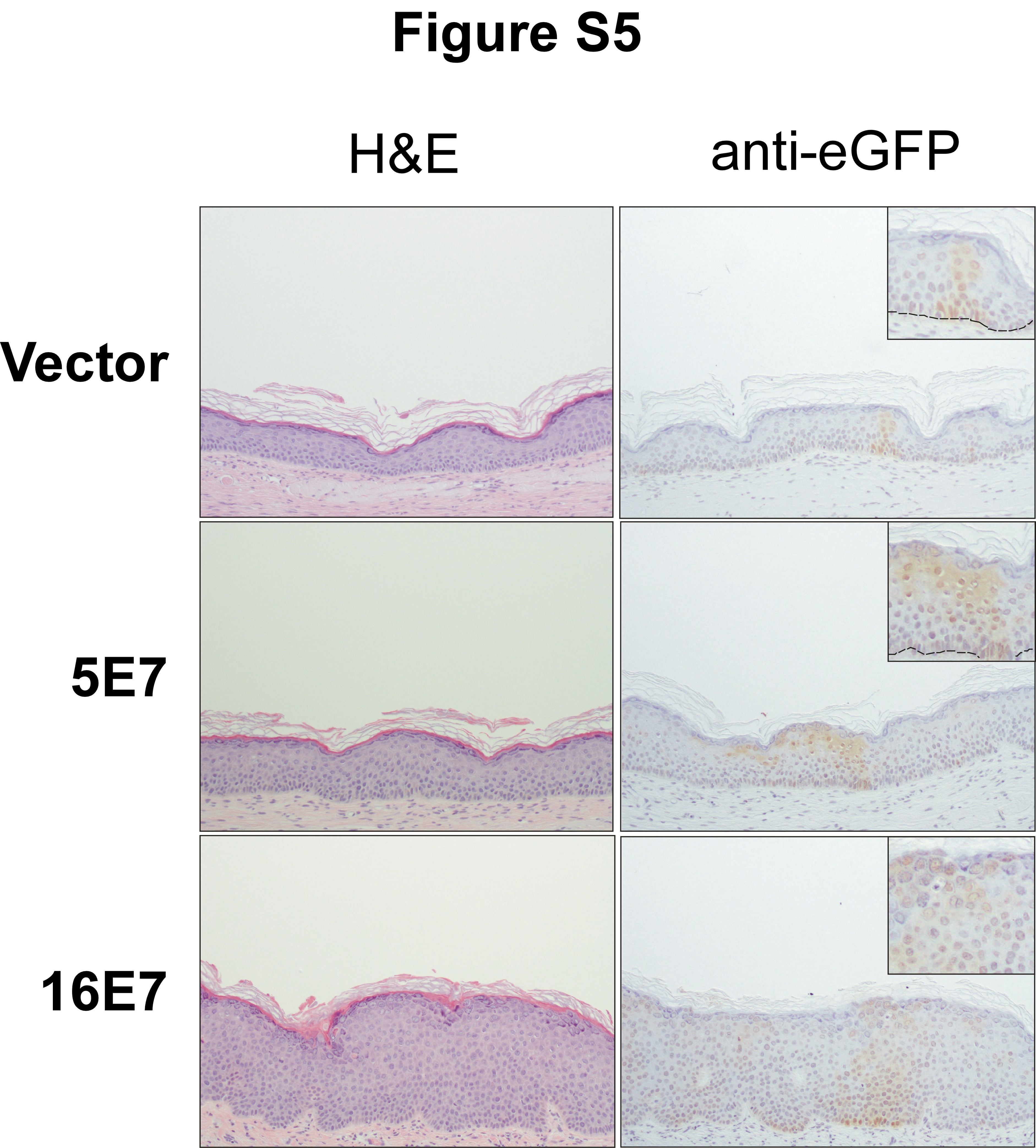

Supplement: Figure S5 — Immunostaining with anti-eGFP. Patchy expression was observed in the grafts corresponding to the control vector, 5E7 and 16E7 samples. H&E staining of similar areas is also shown. (TIF) [file pone.0041743.s005.tif]

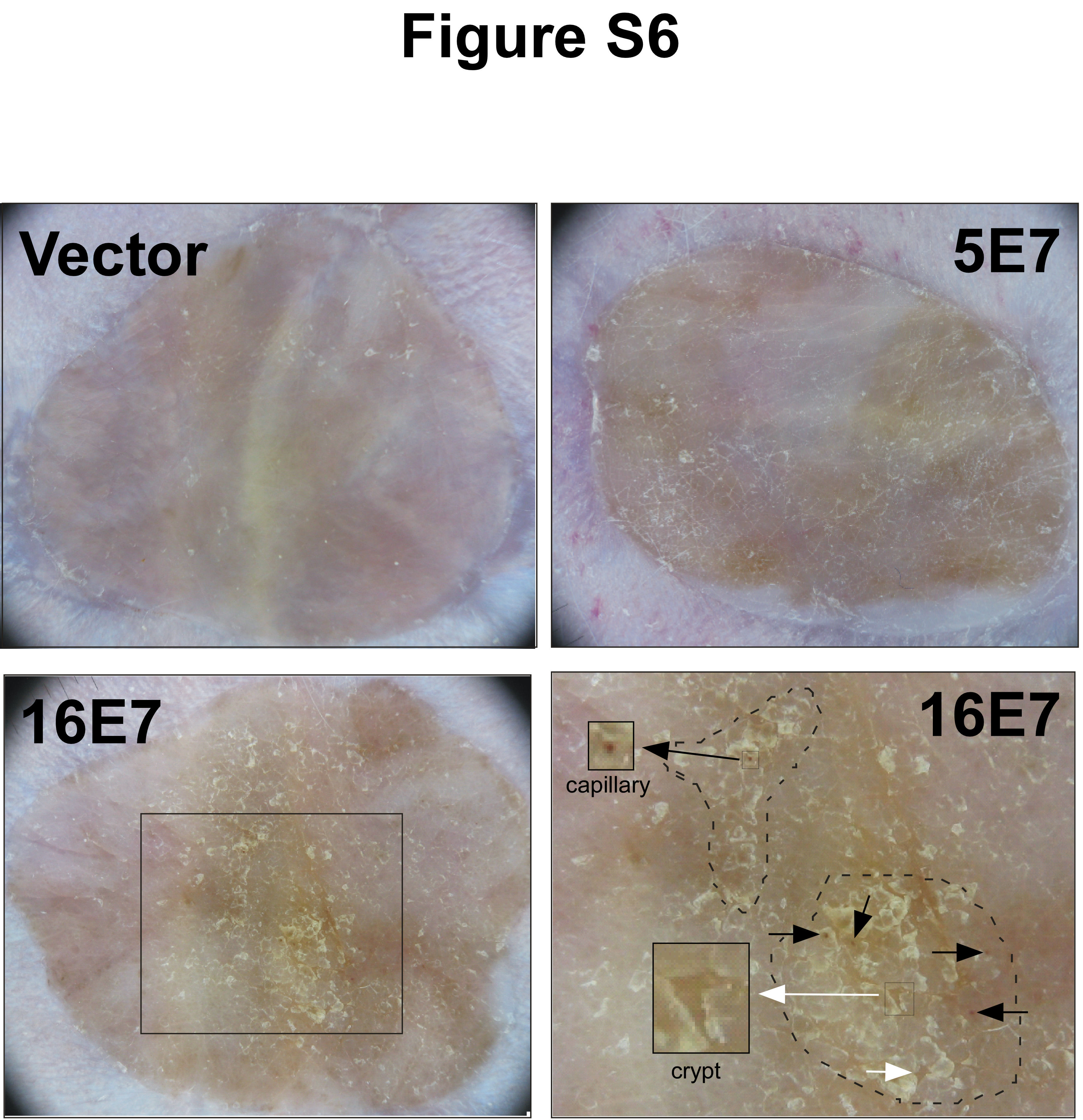

Supplement: Figure S6 — Dermatoscopic images of human skin E7-grafts. Representative images of control vector, 5E7, and 16E7 samples. Lower, right panel of 16E7 represents the highlighted area in the left 16E7 image. Similar features to human viral warts included: i) hyperkeratosis and papillomatosis (areas within dashed lines), ii) capillaries (black arrows, and upper insert) and iii) crypts associated with papillomatosis (white arrows and lower insert). (TIF) [file pone.0041743.s006.tif]

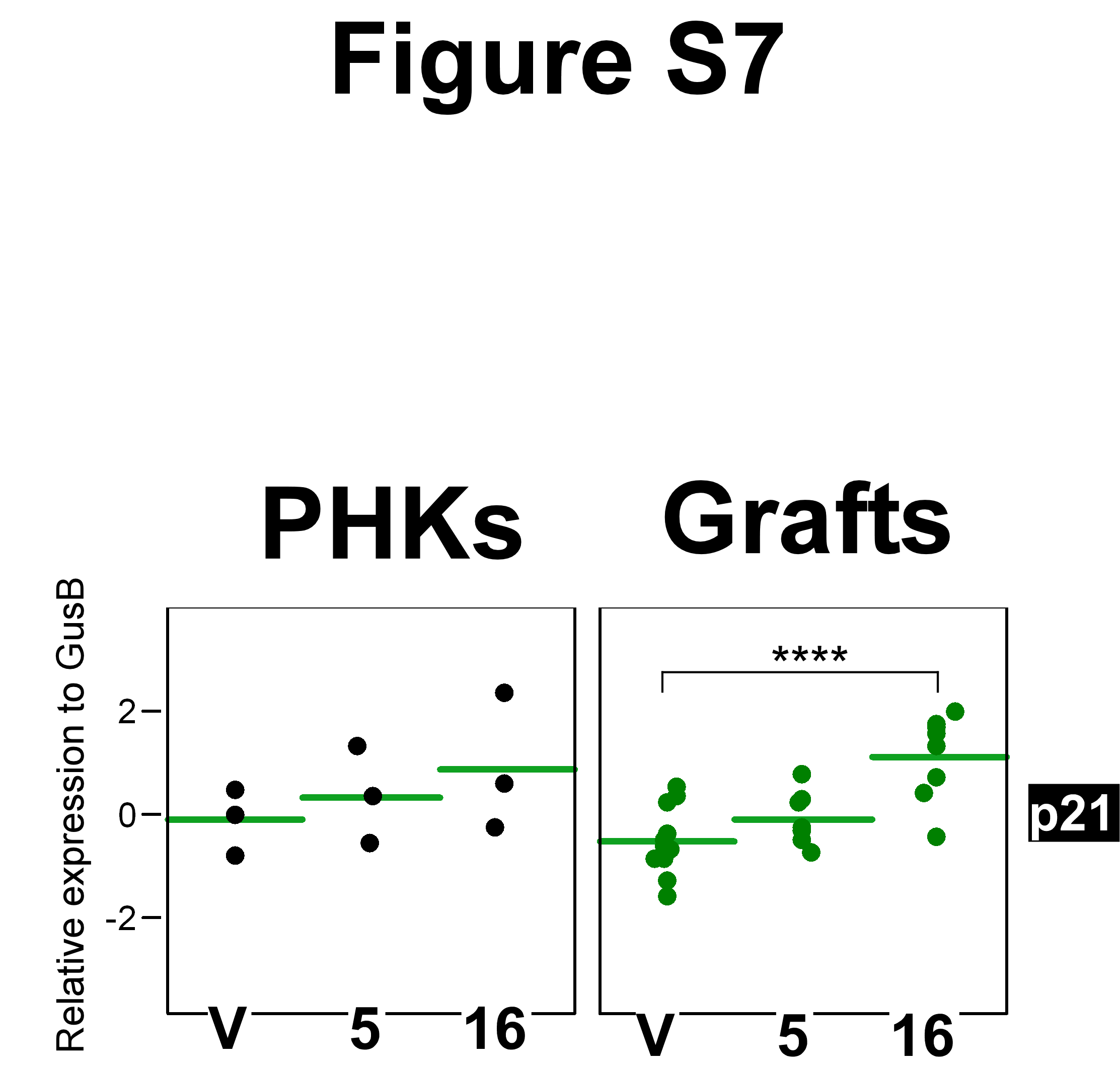

Supplement: Figure S7 — Quantification of p21 mRNA expression. qRT-PCR was conducted on p21, showing a moderate increase in PHK cells before grafting that was significantly augmented after transplantation in the case of HPV16 E7. Each dot represents an individual sample. Horizontal lines represent means for each sample group. Shown are log2-based, z-values of expression relative to housekeeping GUSB in PHK cells and transplants (Materials and Methods). V: control vector; 5: 5E7; 16: 16E7. A Student’s t-test detected significant differences in gene expression between the different samples (threshold p-val<0.05). ****: p-val<0.00005. (TIF) [file pone.0041743.s007.tif]

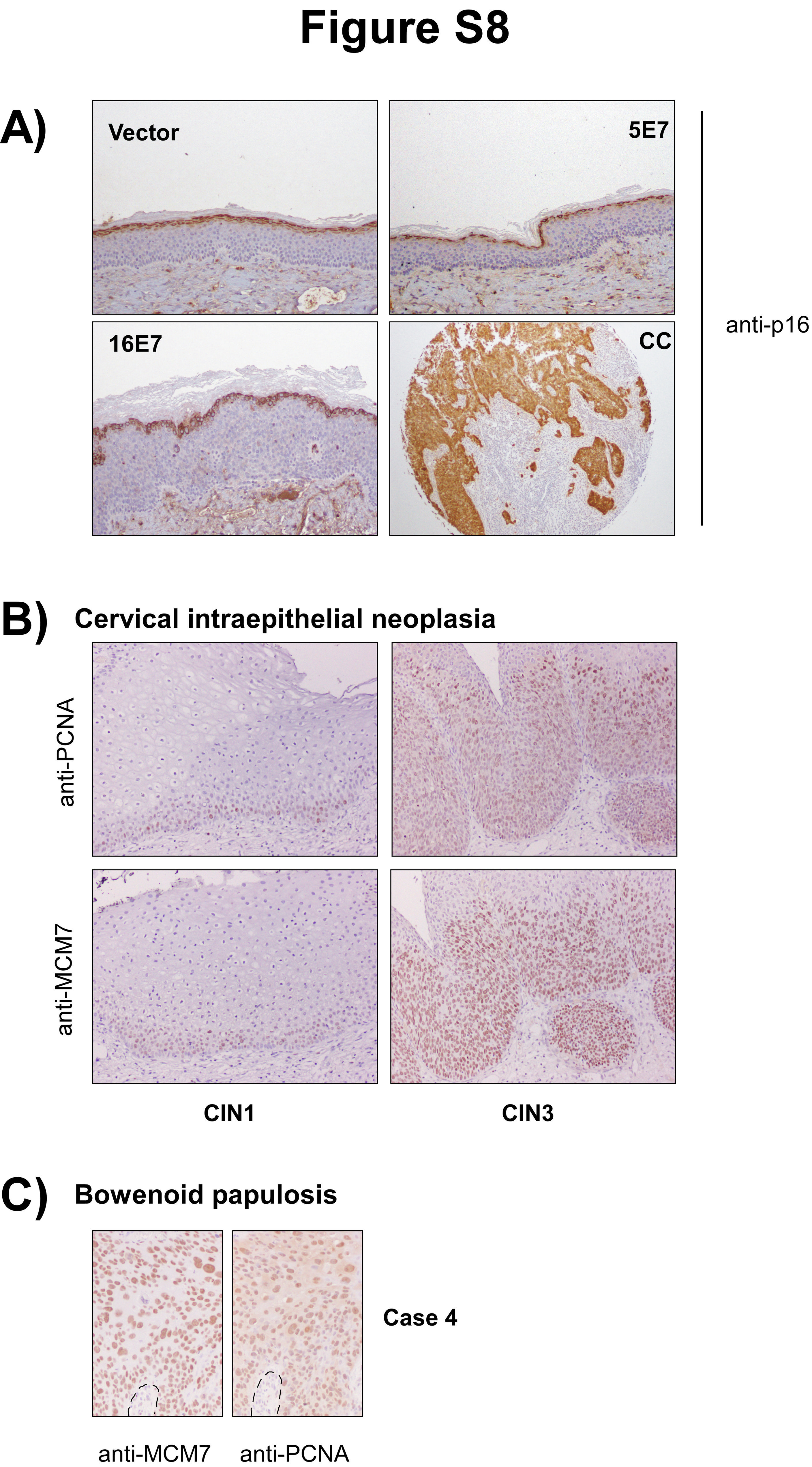

Supplement: Figure S8 — HPV biomarker analysis in human infected anogenital neoplasias. A) Immunohistochemistry of p16 using the CINtec Histology Kit on skin E7-grafts and cervical carcinoma samples. No differences were observed in transplants upon E7 expression. As expected, a CC sample was stained with the p16 antibody. PCNA and MCM7 expression patterns in clinical samples of cervical intraepithelial neoplasia (B) and high grade bowenoid papulosis (C). Dashed lines in C represent the basal membrane. (TIF) [file pone.0041743.s008.tif]

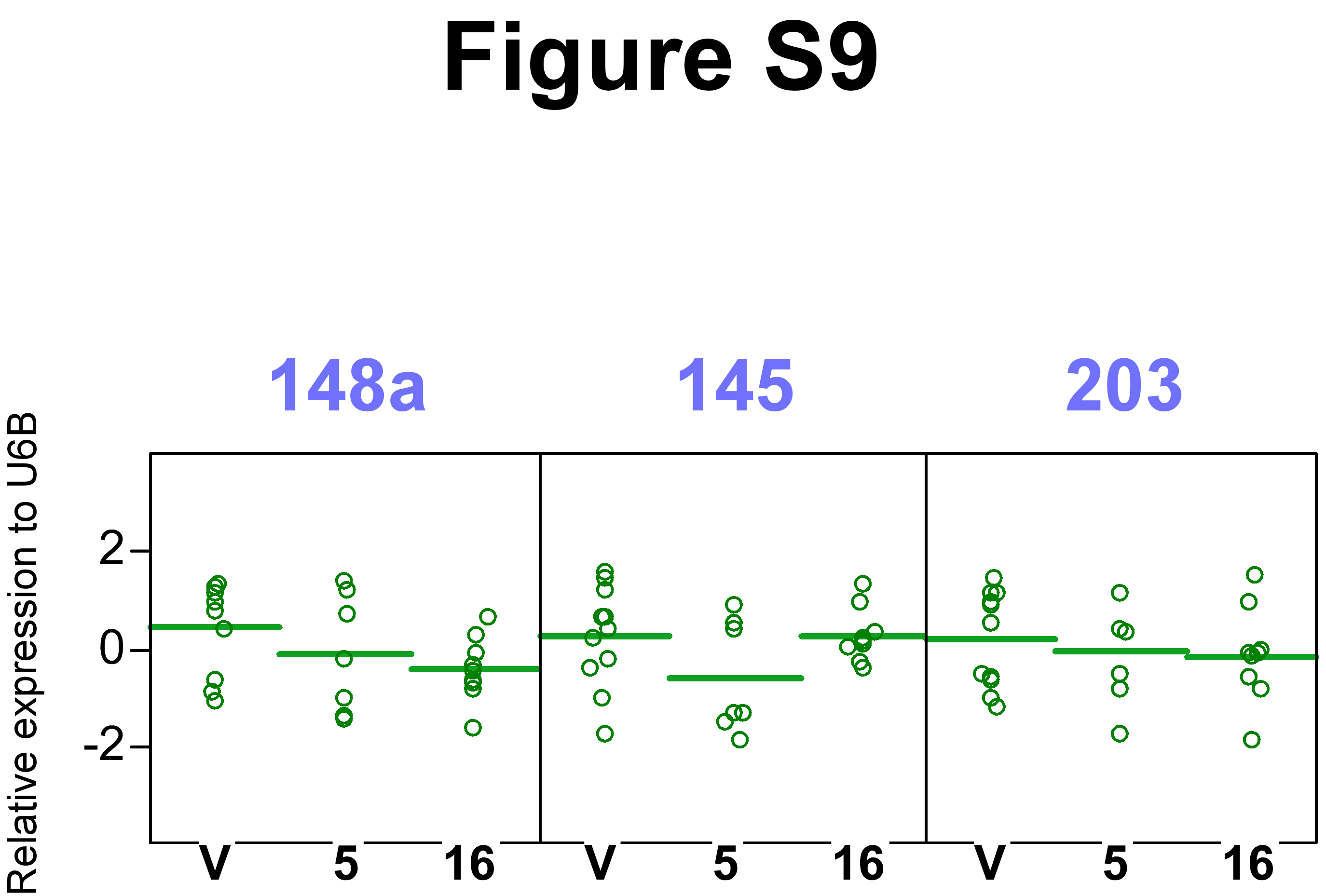

Supplement: Figure S9 — qRT-PCR for miR-148a, miR-145 and miR-203 miRNAs in E7-transplants. Each dot represents an individual sample. Horizontal lines represent means for each sample group. Shown are log2-based, z-values of expression relative to housekeeping U6B (Materials and Methods). V: control vector; 5: 5E7; 16: 16E7. A Student’s t-test was performed to detect significant differences in gene expression between the different samples (threshold p-val<0.05). No significant deregulation was observed. (TIF) [file pone.0041743.s009.tif]
